# 6‐Hydroxydopamine lesion and levodopa treatment modify the effect of buspirone in the *substantia nigra pars reticulata*

**DOI:** 10.1111/bph.15145

**Published:** 2020-07-06

**Authors:** Sergio Vegas‐Suárez, Clarissa Anna Pisanò, Catalina Requejo, Harkaitz Bengoetxea, Jose Vicente Lafuente, Michele Morari, Cristina Miguelez, Luisa Ugedo

**Affiliations:** ^1^ Department of Pharmacology, Faculty of Medicine and Nursing University of the Basque Country (UPV/EHU) Leioa Spain; ^2^ Autonomic and Movement Disorders Unit, Neurodegenerative Diseases Biocruces Health Research Institute Barakaldo Bizkaia Spain; ^3^ Department of Medical Sciences, Section of Pharmacology University of Ferrara Ferrara Italy; ^4^ Neuroscience Center and National Institute of Neuroscience University of Ferrara Ferrara Italy; ^5^ LaNCE, Department of Neuroscience University of the Basque Country (UPV/EHU) Leioa Spain

## Abstract

**Background and Purpose:**

l‐DOPA‐induced dyskinesia (LID) is considered a major complication in the treatment of Parkinson's disease (PD). Buspirone (5‐HT_1A_ partial agonist) have shown promising results in the treatment of PD and LID, however no 5‐HT‐based treatment has been approved in PD. The present study was aimed to investigate how the *substantia nigra pars reticulata* (SNr) is affected by buspirone and whether it is a good target to study 5‐HT antidyskinetic treatments.

**Experimental Approach:**

Buspirone was studied using *in vivo* single‐unit, electrocorticogram, local field potential recordings along with microdialysis and immunohistochemistry in naïve/sham, 6‐hydroxydopamine (6‐OHDA)‐lesioned or 6‐OHDA‐lesioned and l‐DOPA‐treated (6‐OHDA/l‐DOPA) rats.

**Key Results:**

Local buspirone inhibited SNr neuron activity in all groups. However, systemic buspirone reduced burst activity in 6‐OHDA‐lesioned rats (with or without l‐DOPA treatment), whereas 8‐OH‐DPAT, a full 5‐HT_1A_ agonist induced larger inhibitory effects in sham animals. Neither buspirone nor 8‐OH‐DPAT markedly modified the low‐frequency oscillatory activity in the SNr or synchronization within the SNr with the cortex. In addition, local perfusion of buspirone increased GABA and glutamate release in the SNr of naïve and 6‐OHDA‐lesioned rats but no effect in 6‐OHDA/l‐DOPA rats. In the 6‐OHDA/l‐DOPA group, increased 5‐HT transporter and decreased 5‐HT_1A_ receptor expression was found.

**Conclusions and Implications:**

The effects of buspirone in SNr are influenced by dopamine loss and l‐DOPA treatment. The present results suggest that the regulation of burst activity of the SNr induced by DA loss may be a good target to test new drugs for the treatment of PD and LID.

## Graphical abstract

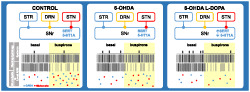

**Abbreviations**
6‐OHDA: 6‐hydroxydopamine
AIMs: Abnormal involuntary movements
ALO: axial, limb, and orolingual
CV: Coefficient of variation
DA: dopamine
ECoG: Electrocorticogram
Glu: glutamate
LFP: Local field potential
LID: l‐DOPA‐induced dyskinesia
PD: Parkinson's disease
SERT: 5‐HT transporter
SNr: *Substantia nigra pars reticulata*
5‐HT: Serotonin

**What is already known**

- Buspirone is effective in reducing l‐DOPA‐induced dyskinesia (LID).
- Neuronal activity and low‐frequency oscillatory activity are increased in the SNr of 6‐hydroxydopamine (6‐OHDA)‐lesioned rats.

**What this study adds**

- Buspirone reduces SNr‐burst activity and amino acid release more markedly after dopamine (DA) loss.
- Expression of 5‐HT_1A_ receptors is reduced and 5‐HT transporter is enhanced in 6‐OHDA/l‐DOPA rats

**What is the clinical significance**

- The findings shed light on the mechanism of action of buspirone in parkinsonism and LID.

## 1 INTRODUCTION

Parkinson's disease (PD) pathophysiology involves the progressive loss of different peripheral and central neuronal populations. Degeneration of dopaminergic neurons of the *substantia nigra pars compacta*, leading to a decrease in striatal dopamine (DA) levels, underlies the appearance of motor deficits (Kish, Shannak, & Hornykiewicz, 1988). The current treatment of Parkinson's disease motor symptoms focuses on restoring dopaminergic neurotransmission using the DA precursor l‐ DOPA, dopaminergic receptor agonists and DA metabolism inhibitors. l‐DOPA provides the greatest symptomatic benefit, although its prolonged use is associated with motor complications and decreased efficacy (Olanow, 2008). The appearance of abnormal involuntary movements, known as l‐DOPA‐induced dyskinesia (LID), complicates the management of motor symptoms and, in some cases, can greatly affect patient quality of life. Only amantadine (an N‐methyl‐D‐aspartate (NMDA) receptor antagonist) is currently marketed as antidyskinetic, but it lacks long‐term efficacy (Perez‐Lloret & Rascol, 2018).

Many drugs acting on 5‐HT receptors, such as 5‐HT_1A_ agonists, 5‐HT_1A/B_ agonists, 5‐HT_2A_ antagonists or 5‐HT transporter (SERT) inhibitors have been tested and proven to be effective in reducing l‐DOPA‐induced dyskinesia in animal models (Bibbiani, Oh, & Chase, 2001; Bishop et al., 2012; Grégoire et al., 2009; Hamadjida et al., 2018; Lindgren, Andersson, Lagerkvist, Nissbrandt, & Cenci, 2010). However, no 5‐HT‐based therapy has been approved for clinical use. The main problem is that, regardless of the preclinical anti‐dyskinetic evidence, none of these drugs have achieved good enough results in clinical trials.

In spite of this lack of clinically efficacy of 5‐HT drugs, several data support the therapeutic relevance of the 5‐HT system in l‐DOPA‐induced dyskinesia as, for example, the fact l‐DOPA is taken up, metabolized to dopamine and released from 5‐HT nerve terminals (for review, see Miguelez, Benazzouz, Ugedo, & De Deurwaerdère, 2017; Politis et al., 2014). Furthermore, results from studies in patients with Parkinson's disease show a correlation between 5‐HT_1A_/SERT binding sites and tremors (Caretti et al., 2008; Doder, Rabiner, Turjanski, Lees, & Brooks, 2003; Loane et al., 2013; Qamhawi et al., 2015) as well as a relationship between post‐mortem SERT binding and dyskinesia‐free survival time (Rylander et al., 2010).

When investigating the possible effect of antidyskinetic drugs, it is important to take into account the results collected in animal models of Parkinson's disease that reveal the structural and functional changes in the 5‐HT system induced by DA depletion and/or chronic administration of l‐DOPA (see review Vegas‐Suarez et al., 2019). In this regard, we have observed that buspirone, a 5‐HT_1A_ receptor partial agonist, inhibits neuronal activity in the subthalamic nucleus in control rats but has no effect after DA depletion (Sagarduy et al., 2016). Another study found that it efficiently improves the behavioural score and reduces certain molecular changes associated with l‐DOPA‐induced dyskinesia (Azkona et al., 2014). In addition, DA depletion and/or chronic administration of l‐DOPA have been associated with dysfunctional neuronal activity in several brain structures among which is the *substantia nigra reticulata* (SNr) (Aristieta, Ruiz‐Ortega, Miguelez, Morera‐Herreras, & Ugedo, 2016; Tseng, Riquelme, Belforte, Pazo, & Murer, 2000). The SNr, one of the main basal ganglia output nuclei, is densely innervated by 5‐HT nerve fibres and contains 5‐HT and 5‐HT_1A_ receptors (see Di Matteo et al., 2008). Furthermore, electrical stimulation targeting the SNr alleviates some Parkinson's disease symptoms (Collomb‐Clerc & Welter, 2015; Hidding et al., 2019; Weiss et al., 2013), while striatonigral optostimulation induces dyskinesia in mice (Keifman et al., 2019).

The present study aimed at defining whether the SNr is involved in the effects of buspirone and that it might be a good target to investigate antidyskinetic drugs. For this purpose, electrophysiological, immunohistochemical and neurochemical approaches were applied in 6‐hydroxydopamine (6‐OHDA)‐lesioned rats with and without prolonged treatment with l‐DOPA.

## 2 METHODS

### 2.1 Animals

Animal studies are reported in compliance with the ARRIVE guidelines (Kilkenny, Browne, Cuthill, Emerson, & Altman, 2010) and with the recommendations made by the *British Journal of Pharmacology.* For electrophysiological and immunohistochemical experiments, male Sprague–Dawley rats (SGIker facilities, University of the Basque Country, UPV/EHU, Spain) weighing 150–175 g at the beginning of the experiments were housed in groups of at least four animals under standard laboratory conditions (22 ± 1°C, 55 ± 5% relative humidity and a 12:12‐h light/dark cycle) with ad libitum access to food and water. Experiments involving animals were approved by the Local Ethical Committee of the University of Basque Country (UPV/EHU, CEEA/M20/2016/176) following European (2010/63/UE) and Spanish (RD 53/2013) regulations for the care and use of laboratory animals.

Microdialysis experiments were performed using male Sprague–Dawley rats (150 g; Charles River, Calco, Lecco, Italy) following protocols approved by the Ethical Committee of the University of Ferrara and the Italian Ministry of Health (license no. 714/2016‐PR). Every effort was made to minimize animal suffering and to use the minimum number of animals per group and experiment.

### 2.2 Experimental design

**FIGURE 1:**
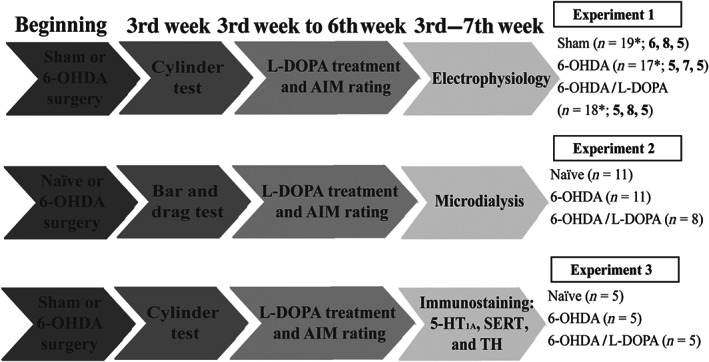
Schematic representation of the experimental design. At the beginning of the three experiments, animals received a vehicle or 6‐OHDA injection into the right medial forebrain bundle. Three weeks later, dopaminergic depletion was evaluated by different asymmetry tests (cylinder or bar and drag test). After that, rats received a 21‐day treatment of daily l‐DOPA injections (6 plus 12 mg·kg^−1^ benserazide). Abnormal involuntary movements (AIMs) were scored at baseline and at the end of the treatment. In experiments 1 and 2, electrophysiological recordings and microdialysis assays in the SNr were performed after the l‐DOPA chronic treatment. Finally, animals were perfused transcardially and processed for histological examination. In experiment 3, sacrifice and tissue processing was performed after chronic treatment. ^*^The group was divided into three subgroups with the respective sizes indicated by bold numbers. TH, tyrosine hydroxlase

Experiments were performed and results were analysed under blinded conditions according to *British Journal of Pharmacology* (Kilkenny et al., 2010). A total of 105 rats were used in this study (six rats were excluded for technical reasons). Animals were divided into three groups which are referred to as sham/naïve, 6‐OHDA and 6‐OHDA/l‐DOPA groups. Animals were randomly assigned to each group and treatment. Only male animals were used, in order to avoid sex variability. Initially, all groups had equal size, but after behavioural test or immunohistochemistry verification of dopamine loss, results from six animals were excluded. The size of each group for every study design was determined by considering the accuracy and reproducibility of methods used according to our previous published work. Figure 1 shows the timeline and the number of animals (in brackets) used in each experiment. For electrophysiology recordings (experiment 1) and for 5‐HT_1A_ and SERT immunostaining (experiment 3), rats received 6‐OHDA or vehicle injections into the medial forebrain bundle and the severity of the dopaminergic lesion was screened using the cylinder test at least 2 weeks later. l‐DOPA was then administered daily for 3 weeks and abnormal involuntary movements (AIMs) were evaluated at baseline and on the 21st day. Electrophysiological recordings or perfusion were performed at least 3 weeks after the lesion (sham or 6‐OHDA group) or 24 h after the last dose of l‐DOPA (6‐OHDA/l‐DOPA‐treated group). At the end of the electrophysiological experiments, animals were deeply anaesthetized (1.2 g·kg^−1^ urethane, i.p.), perfused with saline followed by 4% ice‐cold paraformaldehyde prepared in 0.1‐M phosphate buffer and brains removed for histological verification. For microdialysis experiments (experiment 2), rats received 6‐OHDA injections and were screened using the bar and drag tests 3 weeks later. l‐DOPA was administered for 3 weeks and abnormal involuntary movements were evaluated. At least 3 weeks after the lesion or 24 h after last l‐DOPA injection, rats were implanted with microdialysis probes. Each animal underwent two microdialysis sessions (24 and 48 h after surgery) and received Ringer solution or buspirone administration in a randomized fashion. Samples were collected every 20 min for a total of 4 h. At the end of the experiment the animals were killed by isoflurane overdose and the brains removed and stored at −80°C. Coronal sections (40 μm) were cut using a cryostat LEICA microtome and the correct placement of the microdialysis probe within the SNr was verified.

### 2.3 6‐OHDA lesions

6‐OHDA lesions were performed according to our established protocols (Aristieta et al., 2016). Thirty minutes before surgery, rats were pretreated using desipramine (25 mg·kg^−1^, i.p.) and pargyline (50 mg·kg^−1^, i.p.) in order to preserve the noradrenergic system and the degradation of the toxin, respectively. Then rats were deeply anaesthetized using isoflurane (1.5–2%, Esteve) and placed in the stereotaxic frame (David Kopf® Instruments). 6‐OHDA (3.5 μg·μl^−1^, in 0.02% ascorbic acid) or vehicle was injected using a 10‐μl Hamilton syringe at a rate of 1 μl·min^−1^. A total of 7.5 and 6 μl, respectively, was injected at two sites in the right medial forebrain bundle—2.5 μl at anteroposterior (AP): −4.4 mm, mediolateral (ML): +1.2 mm and dorsoventral (DV): −7.8 mm relative to bregma and dura with the toothbar set at −2.4 and 2 μl at AP: −4.0 mm, ML: +0.8 mm and DV: −8 mm with toothbar at +3.4 (Paxinos & Watson, 1997). The parkinsonian animals used in experiment 2 (see Figure 1) were lesioned following the same procedure with slight modifications according to Arcuri et al. (2018).

### 2.4 Behavioural tests

#### 2.4.1 Cylinder test

At least 2 weeks after the 6‐OHDA lesion, forelimb asymmetry was evaluated using the cylinder test as described previously (Miguelez, Aristieta, Cenci, & Ugedo, 2011). Rats were placed in a transparent glass cylinder (20 cm of diameter) with two mirrors positioned at a 90° angle behind the cylinder to allow a complete view of the exploratory activity. Each animal was left in place, freely exploring, until it made at least 20 weight‐bearing contacts on the glass walls using either forelimb (maximal exploration time of 5 min). The percentage of contralateral forelimb use with respect to the total number of contacts was calculated. Only animals with an ipsilateral contact percentage ≥80% were enrolled in the experiments 1 or 3.

#### 2.4.2 Bar test

Three weeks after the 6‐OHDA lesion, the bar test was performed as previously described (Arcuri et al., 2018). The ipsilateral and contralateral forelimbs were alternatively placed on three blocks of increasing heights (3, 6 and 9 cm). The immobility time (in seconds) of each forelimb on the blocks was recorded for each step. Only animals that reached a 20‐s immobility time with the contralateral forelimb were included in experiment 2.

#### 2.4.3 Drag test

Three weeks after the 6‐OHDA lesion, the drag test was performed (Arcuri et al., 2018). Briefly, each rat was lifted from the abdomen, leaving each forelimb on the table and dragged backwards along a fixed distance of 1 m at a constant speed of 20 cm·s^−1^. Two different observers counted the number of contacts made by each forelimb. Animals with a percentage of ipsilateral contacts with respect to total contacts ≥80% were included in experiment 2.

#### 2.4.4 Long‐term l‐DOPA treatment and abnormal involuntary movements rating

Abnormal involuntary movements were induced in 6‐OHDA‐lesioned rats by daily injections of l‐DOPA (6 mg·kg^−1^, i.p.) in combination with the peripheral decarboxylase inhibitor benserazide (12 mg·kg^−1^, i.p.) over 3 weeks (Aristieta et al., 2016; Aristieta, Ruiz‐Ortega, Morera‐Herreras, Miguelez, & Ugedo, 2019; Azkona et al., 2014; Dekundy, Lundblad, Danysz, & Cenci, 2007). Abnormal involuntary movements were scored according to the scale described by Cenci and Lundblad (2007). On the testing days, rats were placed individually in transparent empty plastic cages for at least 10 min before drug administration. Each rat was observed for one full minute every 20th minute along the 200‐min testing period. The severity of the three subtypes of dyskinetic movements (axial, limb and orolingual [ALO]) were rated from 0 to 4 based on the amount of time for which the abnormal movement was observed (i.e. 0, not present to 4, continuous). In addition, the amplitude of ALO abnormal involuntary movements were rated on a scale from 0 to 4 separately from locomotive abnormal involuntary movements. Data are expressed as the abnormal involuntary movements score/session for ALO, which were calculated by multiplying the severity score by the amplitude scores during the monitoring period, with all of these products summed for each testing session (Lindgren et al., 2010). All 6‐OHDA l‐DOPA rats developed severe dyskinetic movements that peaked between 40 and 100 min after a single injection of l‐DOPA and had a total score >100 on the 21st treatment day (Figure S1).

### 2.5 Electrophysiological procedures

The electrophysiological recordings of SNr neurons were performed at least 3 weeks after 6‐OHDA or vehicle injection for the sham or 6‐OHDA group or 24 h after the last l‐DOPA injection in the 6‐OHDA l‐DOPA group (Aristieta et al., 2016). Rats were anaesthetized using urethane (1.2 g·kg^−1^, i.p.) and the right jugular vein was cannulated for systemic drug administration. Next, each rat was placed in a stereotaxic frame with its head secured in a horizontal orientation. The rats were allowed to breath spontaneously. The recording electrode was lowered into the right SNr (relative to bregma and dura, AP: −5.3 mm, ML: −2.5 mm, DV: −7.5 to 8.5 mm) (Paxinos & Watson, 1997). Neuronal spikes were digitized using the CED micro 1401 interface and Spike2 software (Cambridge Electronic Design). The basal firing rate was recorded for 300 s and at least 150 s after each systemic drug dose. For local administration, a calibrated pipette glued adjacent to a recording micropipette was filled with buspirone 0.25 M dissolved in Dulbecco's buffered saline solution (NaCl 136.9 mM; KCl 2.7 mM; NaH_2_PO_4_ 8.1 mM; KH_2_PO_4_ 1.5 mM; MgCl_2_ 0.5 mM; and CaCl_2_ 0.9 mM, pH 7.4) as previously performed by Sagarduy et al. (2016). Local buspirone injection in the SNr was applied with pressure pulses (50–150 ms) using a Picospritzer™ II (General Valve Corporation, Fairfield, NJ, USA). The injected volume was simultaneously monitored with soft movements in a calibrated pipette in which each pulse corresponded to the injection of 2 nl of solution. Several ejection pulses were applied in each application. The effect of each dose on firing rate was recorded until the neuron recovered. At the end of the experiments, a 5‐μA cathodal current was passed through the recording electrode to deposit a discrete mark of Pontamine Sky on the recording site.

The electrocorticogram (ECoG) was simultaneously recorded via a 1‐mm‐diameter steel screw, which was juxtaposed to the dura mater above the right frontal somatic sensory‐motor cortex (AP: +4.5 mm and ML: −2.5 mm to bregma) (Paxinos & Watson, 1997) as described by Aristieta et al. (2016). The signal was pre‐amplified (10×), amplified (200×) and bandpass filtered (0.1–1000 Hz) in an amplifier (Cibertec S.A., model amplifier 63AC). The discriminated electrocorticogram activity (sampled at 2500 Hz) was digitized, stored and analysed using computer software. The local field potential (LFP) in the SNr was recorded through the same glass electrodes that were used for single‐unit extracellular recordings. The signal was pre‐amplified (10×) and then amplified (10×) in a high‐input impedance amplifier (Cibertec S.A., model amplifier AE‐2) where the signal was also bandpass filtered (0.1–5000 Hz). This signal was divided into two different signals in a second amplifier (Cibertec S.A., model amplifier 63AC), namely, the single‐unit extracellular signal and the local field potential signal. In this second amplifier, the local field potential signal was amplified (10×) and bandpass filtered (0.1–100 Hz). The discriminated local field potential activity (sampled at 2500 Hz) was digitized, stored and analysed using computer software.

#### 2.5.1 Analysis of electrophysiological data

All electrophysiological parameters were analysed off‐line using the Spike2 software (version 7). Firing patterns were determined by analysing the interspike interval histogram:‐ firing rate and the coefficient of variation (CV), which represents the measure of firing regularity and is defined as the percentage ratio of the SD to the mean interval histogram, which is obtained applying a Spike2 script (“meanix.s2s”). The percentage of neurons exhibiting burst firing pattern from the total and the burst‐related parameters: the number of bursts (total number of burst detected in the spike train), mean duration of burst (mean duration of the total burst detected in the spike train and expressed in ms), spikes per burst (mean of number of burst detected), recurrence of burst (number of burst per min) and intraburst frequency (mean of the interval of spike patterns within burst) were analysed during a period of 90 s applying a Spike2 script (“surprise.s2s”) based on Poisson Surprise method. For oscillatory activity and synchronization parameters analysis, local field potential and electrocorticogram signals (sampled at 2500 Hz) were smoothed to 1 ms and SNr action potentials were converted to a series of events (sampled at 2500 Hz). These series of events were then transformed to a continuous waveform (1‐ms smoothing period) using a custom‐made Spike2 software script. The power spectrum of smoothed local field potentials, electrocorticograms and SNr spike waveforms and the coherence analyses between SNr spike waveforms and local field potentials, SNr spike waveforms and electrocorticograms, and SNr‐local field potential and electrocorticograms were also analysed using the fast Fourier transform (8,192 blocks size) in the low‐frequency range (0–5 Hz) from 90 s of data applying a Spike2 script (“coherac90.s2s”). The significance of the coherence was determined by the equation described by Rosenberg, Amjad, Breeze, Brillinger, and Halliday (1989): 1 − (1 − *α*)1/(*L* − 1), where *α* is 0.95 and *L* is the number of windows used. The AUC of coherence and power spectrum curves were calculated in the 0‐ to 5‐Hz low‐frequency range.

### 2.6 Probe implantation and *in vivo* microdialysis procedures

Naïve, 6‐OHDA and 6‐OHDA/l‐DOPA rats were stereotaxically implanted with one microdialysis probe in the right SNr under isoflurane anaesthesia (Morari, O'Connor, Ungerstedt, Bianchi, & Fuxe, 1996) according to the following coordinates relative to bregma and dura—AP: −5.5 mm, ML: −2.2 mm and DV: −8.7 mm (Paxinos & Watson, 1997). Concentric microdialysis probes were constructed using AN69 (Gambro Industries, Meyzieu, France) semipermeable hollow membranes (65‐kDa MW cut‐off 340‐μm outer diameter with an active surface of 1.0 mm). Twenty‐four hours after surgery, the experimet was performed in conscious‐freely moving rats. Probes were thoroughly flushed (3.0 μl·min^−1^) with a modified Ringer solution (CaCl_2_ 1.2 mmol·L^−1^, KCl 2.7 mmol·L^−1^, NaCl 148 mmol·L^−1^ and MgCl_2_ 0.85 mmol·L^−1^). After 6 h of rinsing, four baseline samples were collected (every 15 min) and buspirone was perfused at three increasing concentration levels (50, 150 and 500 nM) for 1 h at each concentration. A second microdialysis session was repeated 48 h after surgery. Rats were randomized to Ringer/Ringer or Ringer/buspirone in the first session and then treatment was crossed in the following session.

#### 2.6.1 Analysis of endogenous GABA and Glutamate

Glutamate (Glu) and GABA were measured using HPLC coupled with fluorometric detection as previously described (Bido, Marti, & Morari, 2011). Thirty microlitres of o‐phthaldialdehyde/mercaptoethanol reagent was added to 30‐μl aliquots of sample and 50 μl of the mixture was automatically injected (Triathlon autosampler; Spark Holland, Emmen, Netherlands) onto a 5‐C18 Hypersil ODS analytical column (3‐mm inner diameter, 10‐cm length; Thermo Fisher Scientific, Waltham, MA) perfused at a flow rate of 0.48 ml·min^−1^ (Jasco PU‐2089 Plus quaternary pump; Jasco, Tokyo, Japan) with a mobile phase containing 0.1‐M sodium acetate, 10% methanol and 2.2% tetrahydrofuran (adjusted to pH 6.5 with glacial acetic 99%). Glutamate and GABA were detected by means of a fluorescence spectrophotometer (FP‐2020 Plus; Jasco, Tokyo, Japan) managed by the ChromNAV 2.0 HPLC software (Jasco, MD, USA). The excitation and the emission wavelengths were set at 370 and 450 nm, respectively. Under these conditions, the limits of detection for glutamate and GABA were ~1 nM (i.e., ~147 pg·ml^−1^) and ~0.5 nM (i.e., ~51 pg·ml^−1^) and maximum sensitivity was ~10 μM for both amino acids. The retention times were between 2.8 and 4 min and between 16 and 19 min for glutamate and GABA, respectively.

### 2.7 Histological procedures and analysis

Animals were deeply anaesthetized (1.2 g·kg^−1^ urethane i.p.) and transcardially perfused with saline followed by 4% ice‐cold paraformaldehyde and 0.2% picric acid prepared in 0.1‐M phosphate saline buffer. Brains were removed and the following day they were transferred to a 25% sucrose solution until they sank. The brains were serially cut in coronal 40‐μm sections using a freezing microtome and slices were conserved in a cryoprotectant solution at −20°C until further processing.

#### 2.7.1 Immunohistochemistry

The Immuno‐related procedures used comply with the recommendations made by the *British Journal of Pharmacology* (Alexander et al., 2018). For tyrosine hydroxylase (TH) immunochemistry, sections containing the striatum and the SNc were washed in potassium PBS (KPBS; 0.02 M, pH 7.4). Then endogenous peroxidase was quenched using 3% H_2_O_2_ and 10% (v/v) methanol in KPBS for 30 min at 22°C. After rinsing in KPBS, brain sections were preincubated in 1% Triton X‐100 with KPBS (KPBS‐T) and 5% normal goat serum for 1 h at 22°C and then incubated with primary antibody (rabbit anti‐TH, AB152, 1:1,000, Merck Millipore, Spain) overnight at 22°C. Afterwards, the sections were incubated with secondary antibody (biotinylated goat anti‐rabbit IgG, BA‐1000, 1:200, Vector Laboratories, California, USA) in KPBS‐T with 2.5% NGS for 2 h at RT. All sections were incubated for 1 h with an avidin–biotin–peroxidase complex (ABC kit, PK‐6100, Vector Laboratories) as chromogen and peroxidase activity was visualized using 0.05% 3,3′‐diaminobenzidine (DAB) and 0.03% H_2_O_2_ for 1–2 min. The reaction was stopped by rinsing in KPBS and sections were mounted onto gelatin‐coated slides, dehydrated in ascending series of ethanol, cleared in xylene and coverslipped using DPX mounting medium.

For SERT and 5‐HT_1A_ receptor immunohistochemistry, sections containing the SNr were first rinsed in phosphate buffer (PB; 0.1 M, pH 7.4) and then incubated for 30 min in 30% methanol and 3% H_2_O_2_ in 0.1‐M PB. After rinsing with PB, sections were placed in 1% sodium borohydride for 30 min. The sections were profusely washed with PB before rinsing in Trizma base saline (TS; 0.1 M, pH 7.6) and then incubated in 0.5% BSA and 0.25% Triton (X‐100) in TS (TS‐T) for 30 min. Then they were incubated with primary antibody (rabbit anti‐SERT, 1:2,500, 24330, Immunostar, Hudson, WI, USA; or rabbit anti‐5‐HT_1A_, 1:200, GTX104703, Genetex, California, USA) for 48 h at 22°C. Later, the sections were incubated with the secondary biotinylated antibody (donkey anti‐rabbit IgG, 1:400, 711‐005‐152, Jackson Immunoresearch, Stratech Scientific; or goat anti‐rabbit IgG, BA‐1000, 1:200, Vector Laboratories) in TS‐T with 0.5% BSA for 2 h at 22°C. Sections were rinsed in TS followed by incubation for 1 h in avidin–biotin–peroxidase complex. The reaction was visualized using 0.022% DAB and 0.003% H_2_O_2_ for 10–15 min, as described previously (Aristieta et al., 2012). The reaction was stopped by rinsing in TS and PB. Finally, sections were mounted, dehydrated and coverslipped.

#### 2.7.2 Integrated optical densitometry of the SNr

The integrated optical densitometry (IOD) of tyrosine hydroxylase in the striatum and SERT and 5‐HT_1A_ receptors in the SNr were measured using NIH‐produced software, ImageJ win64 Fiji (https://imagej.net/Fiji) for reading ODs as grey levels. Digital images from sections were obtained with the 20× objective of an automatic panoramic digital slide scanner (Pannoramic MIDI II, 3DHistech, Hungary) using the CaseViewer 2.3 (64‐bit version) software. One blinded investigator performed the integrated optical densitometry reading. The mean integrated optical densitometry in the SNr was determined by subtracting the background for each section. Measurements were performed for two to four sections throughout the SNr and the mean per animal was calculated. Results (IOD%) were expressed as the percentage of optical intensity of the ipsilateral lesioned hemisphere with respect to the intact or contralateral non‐lesioned hemisphere.

### 2.8 Drugs

The following drugs were used in this study: urethane, 6‐hydroxydopamine hydrobromide, desipramine hydrochloride, pargyline, l‐DOPA, benserazide hydrochloride and buspirone hydrochloride, which were obtained from Sigma‐Aldrich. WAY‐100635 maleate and 8‐hydroxy‐2‐(di‐n‐propylamino)‐tetralin (8‐OH‐DPAT) were purchased from Tocris‐Biogen. Desipramine, pargyline, l‐DOPA, benserazide, buspirone, 8‐OH‐DPAT and WAY‐100635 were prepared in 0.9% saline. Urethane was dissolved in Milli‐Q water. 6‐OHDA was dissolved in Milli‐Q water containing 0.02% ascorbic acid. All drug solutions were prepared on the day of the experiment.

### 2.9 Statistical analysis

The data and statistical analysis comply with the recommendations of the *British Journal of Pharmacology* on experimental design and analysis in pharmacology (Curtis et al., 2018). Group size was ≥5, which is the number of independent values. Experimental data were analysed using GraphPad Prism (v. 5.01, GraphPad Software, Inc.). Differences between experimental groups on basal electrophysiological parameters were analysed using one‐way ANOVA except the burst firing parameters, which were compared using the Fisher's exact test. The effect of buspirone or 8‐OH‐DPAT on electrophysiological parameters was matched and analysed by repeated measures (RM) two‐way ANOVA (Lesion × Treatment). As more than one neuron per animal was recorded when evaluating the effect of locally applied buspirone, the electrophysiological parameters values were averaged per animal, so that every animal had one value. In microdialysis, experiments were expressed as a percentage of the basal value (calculated as the mean of two samples before treatment). The effect of buspirone on GABA and glutamate levels in the dialysate was calculated as the summation changes with respect to the baseline (% basal). Basal values among groups were compared using one‐way ANOVA, whereas the effect of buspirone was analysed using RM two‐way ANOVA. For immunostaining results, one‐way ANOVA was used. ANOVAs were followed by Bonferroni's post hoc tests only when it was statistically significant, *P* ≤ 0.05 and there was no significant variance inhomogeneity. The sphericity of RM two‐way ANOVA was assumed by GraphPad Prism software. The threshold of statistical significance was set at *P* < 0.05 with corresponding 95% confidence intervals. Data are presented as mean ± SEM. All statistical details are described in Table S1.

### 2.10 Nomenclature of targets and ligands

Key protein targets and ligands in this article are hyperlinked to corresponding entries in http://www.guidetopharmacology.org, the common portal for data from the IUPHAR/BPS Guide to PHARMACOLOGY (Harding et al., 2018) and are permanently archived in the Concise Guide to PHARMACOLOGY 2019/20 (Alexander et al., 2018).

## 3 RESULTS

### 3.1 Effect of 5‐HT_1A_ drugs on SNr neuronal activity in anesthetized rats

A total of 92 GABAergic neurons from 54 animals were recorded in the SNr of anaesthetized rats—31 from the sham group (*n* = 19 rats), 29 from the 6‐OHDA group (*n* = 17 rats) and 32 from the 6‐OHDA/l‐DOPA group (*n* = 18 rats). All cells recorded were located within the SNr and displayed the characteristic firing properties described for SNr‐GABAergic neurons in our previous publication (Aristieta et al., 2016). The mean value of the basal firing rate was higher in the 6‐OHDA/l‐DOPA group (*P* < 0.05), the coefficient of variation was significantly increased in the 6‐OHDA/group and the number of bursty neurons was significantly increased in the 6‐OHDA and 6‐OHDA/l‐DOPA groups. Mean values ± SEM of the firing rate and firing pattern parameters are shown in Table 1.

**TABLE 1:** Firing properties of neurons of the SNr

	Sham (*n* = 19)	6‐OHDA (*n* = 17)	6‐OHDA/l‐DOPA (*n* = 18)
Firing rate (Hz)	20.9 ± 2.0	26.4 ± 2.0	28.4 ± 2.0^*^
Coefficient of variation (%)	35.7 ± 3.9	53.1 ± 5.6^*^	48.5 ± 5.2
Neurons exhibiting burst firing pattern (%)	41.9	68.9^$^	75.0^$^
Number of bursts	13.7 ± 2.7	25.8 ± 8.9	23.9 ± 9.9
Duration of burst (ms)	0.8 ± 0.2	1.2 ± 0.6	1.2 ± 0.7
Spikes/burst	21.3 ± 7.1	31.6 ± 16.6	57.5 ± 31.8
Recurrence of burst (*n* burst·min^−1^)	8.9 ± 1.7	13.2 ± 4.3	14.6 ± 5.9
Intraburst frequency (spike/s)	49.9 ± 5.7	59.0 ± 6.4	69.1 ± 9.7

*Note*: Data from the firing rate, coefficient of variation, and percentage of neurons exhibiting burst firing pattern (of *n* rats) and burst‐related parameters defined as number of bursts, mean duration of the burst, mean number of spikes per burst, the recurrence of burst per minute, and intraburst frequency or the interval of spike patterns within bursts. Burst activity was detected in the spike train in a period of 90 s based following Surprise method.

^*^ *P* < 0.05 versus sham (one‐way ANOVA followed by Bonferroni's post hoc test).

^$^ *P* < 0.05 versus sham (Fisher's exact test).

**FIGURE 2:**
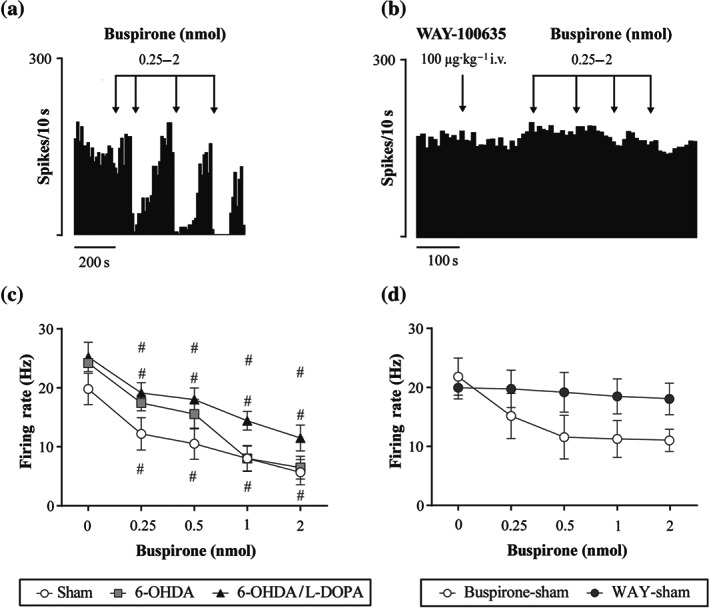
Local administration of buspirone on SNr neuron activity. Representative firing rate histogram illustrating the inhibitory local effect of buspirone on neurons in sham group (a). Representative firing rate histogram illustrating the blockage of the inhibitory local effect of the previous buspirone administration induced by WAY‐100635 (b). Buspirone (0.25–2 nmol) locally injected into the SNr induced a dose‐dependent inhibitory effect in all three experimental groups (c). The inhibitory local effect of buspirone (white circles; *n* = 5 neurons from two sham rats) was blocked by the systemic administration of WAY‐100635 (0.1 mg·kg^−1^, i.v.) (Student's *t*‐test, *P* < 0.05) (grey circle; *n* = 6 neurons from the same two sham rats) (d). Data are expressed as mean ± SEM. ^#^ *P* < 0.05 versus respective baseline (RM two‐way ANOVA followed by Bonferroni's post hoc test) from sham (*n* = 5), 6‐OHDA (*n* = 5), and 6‐OHDA/l‐DOPA (*n* = 5) groups

After recording basal neuronal activity, the experimental groups were divided into three subgroups to study the effect of 5‐HT_1A_ drugs on SNr neuronal activity. The first subgroup animals from sham, 6‐OHDA and 6‐OHDA/l‐DOPA groups (*n* = 6, 5, and 5 rats, respectively) received buspirone locally, the second subgroup animals from control, 6‐OHDA and 6‐OHDA/l‐DOPA groups (*n* = 8, 7, and 8 rats, respectively) received buspirone intravenously, and the third subgroup animals from each group (*n* = 5 rats each) received 8‐OH‐DPAT intravenously. Thus, the local effect of buspirone was studied on 54 SNr neurons from a total of 16 animals: 18 neurons from the sham group (*n* = 6), 17 neurons from the 6‐OHDA group (*n* = 5) and 19 neurons from the 6‐OHDA/l‐DOPA group (*n* = 5). As it is shown in Figure 2a,c, local administration of buspirone (0.25–2 nM) caused a marked, dose‐dependent inhibition of neuronal activity in all three experimental groups, with a maximal reduction of the firing rate of ~70% of the basal value (Figure 2c). No difference in the efficacy of local buspirone was detected when comparing the percentage effect with respect to the basal firing values among the groups (Figure 2c). The inhibitory effect was blocked by previous systemic administration of the 5‐HT_1A_ antagonist WAY‐100635 (100 μg·kg^−1^, i.v.) (*n* = 6 neurons from two control animals, Figure 2b,d).

**FIGURE 3:**
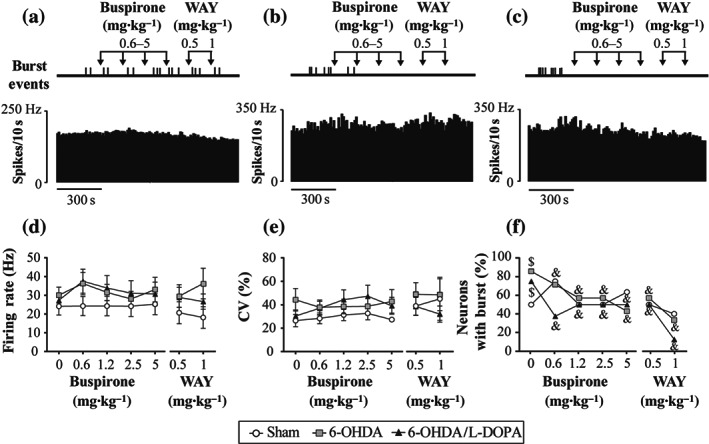
Effect of buspirone on SNr neuron activity. Representative firing rate histogram illustrating the lack of effect of buspirone (0.6125–5 mg·kg^−1^, i.v.) and WAY‐100635 (0.5–1 mg·kg^−1^, i.v.) on SNr neuronal firing rate, and schematic representation of burst firing events that shows the loss of burst firing activity after drug administration in sham (a), 6‐OHDA (b), and 6‐OHDA/l‐DOPA groups (c). Graphical representation of the mean ± SEM of the firing rate (d), the coefficient of variation (CV), (e) and the number of neurons with burst (f) in the three experimental groups, sham (*n* = 8), 6‐OHDA (*n* = 7), and 6‐OHDA/l‐DOPA (*n* = 8) before and after increasing doses of buspirone and WAY‐100635. ^$^ *P* < 0.05 versus sham group and ^&^ *P* < 0.05 versus respective baseline (Fisher's exact test for firing pattern)

Next, the effect of cumulative doses of buspirone (from 0.6125 to 5 mg·kg^−1^, i.v.) on the firing rate and pattern of SNr neurons was studied in 23 rats (sham *n* = 8, 6‐OHDA *n* = 7, and 6‐OHDA/l‐DOPA *n* = 8 animals; Figure 3a–c). One neuron per animal was pharmacologically tested. As shown in Figure 3, systemic administration of buspirone did not modify the firing rate of SNr neurons (Figure 3d) nor the coefficient of variation (Figure 3e) in any of the groups. However, systemic buspirone decreased the number of bursty neurons in the 6‐OHDA and 6‐OHDA/l‐DOPA groups but not in the sham group (*P* < 0.05, Fisher's exact test; Figures 3f and S2). No changes in intraburst parameters were observed. Subsequent intravenous administration of the 5‐HT_1A_ antagonist WAY‐100635 (0.5 and 1 mg·kg^−1^) did not modify either neuron activity or buspirone effect. Table S2 summarizes mean ± SEM values of all firing activity parameters before and after buspirone administration in the three experimental groups.

**FIGURE 4:**
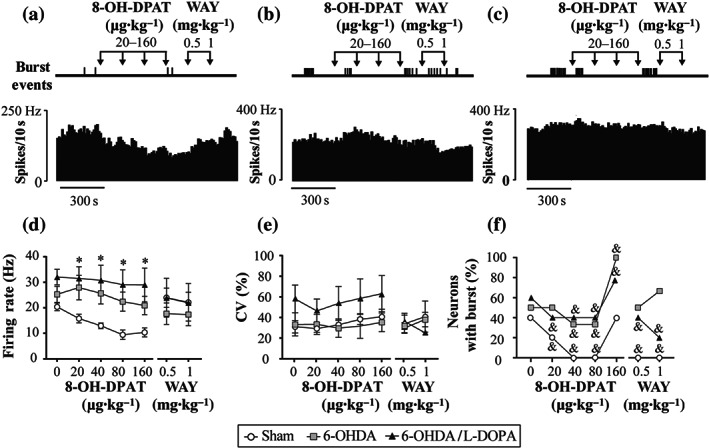
Effect of 8‐OH‐DPAT on SNr neuron activity. Representative firing rate histogram illustrating the absence of effects of 8‐OH‐DPAT (20–160 μg·kg^−1^, i.v.) and WAY‐100635 (0.5–1 mg·kg^−1^, i.v.) on SNr neuronal firing rate, and schematic representation of burst firing events that shows the loss of burst firing activity after drug administration in sham (a), 6‐OHDA (b), and 6‐OHDA/l‐DOPA groups (c). Graphical representation of the mean ± SEM of the firing rate (d), the coefficient of variation (CV). (e) and the number of neurons with burst (f) in the three experimental groups, sham (*n* = 5), 6‐OHDA (*n* = 5), and 6‐OHDA/l‐DOPA (*n* = 5) before and after increasing doses of 8‐OH‐DPAT and WAY‐100635. Data are expressed as mean ± SEM. ^*^ *P* < 0.05 versus sham group (RM two‐way ANOVA followed by Bonferroni's post hoc test), and ^&^ *P* < 0.05 versus corresponding basal value (Fisher's exact test for firing pattern)

Finally, we investigated the effect of systemic administration of 8‐OH‐DPAT (20–160 μg·kg^−1^, i.v.) in 15 animals (sham *n* = 5, 6‐OHDA *n* = 5 and 6‐OHDA/l‐DOPA *n* = 5 animals) (Figure 4a–c). One neuron per animal was pharmacologically tested. A dose‐dependent inhibition of SNr neuron firing rate was observed in the sham group, with a maximal reduction of 47% of the basal value (Figure 4a,d). Conversely, 8‐OH‐DPAT did not modify the firing rate of neurons in 6‐OHDA and 6‐OHDA/l‐DOPA groups (Figure 4d). 8‐OH‐DPAT administration significantly decreased the number of bursty neurons in all three experimental groups (Figure 4f), without modifying intraburst parameters (Table S3) or the coefficient of variation (Figure 4e). WAY‐100635 (0.5–1 mg·kg^−1^, i.v.) partially reversed the effects of 8‐OH‐DPAT (Figure 4f). Table S3 summarizes mean ± SEM values of all firing activity parameters before and after buspirone administration in the three experimental groups.

### 3.2 Effect of 5‐HT_1A_ drugs on the low oscillatory activity and synchronization in the SNr and the cortex

**FIGURE 5:**
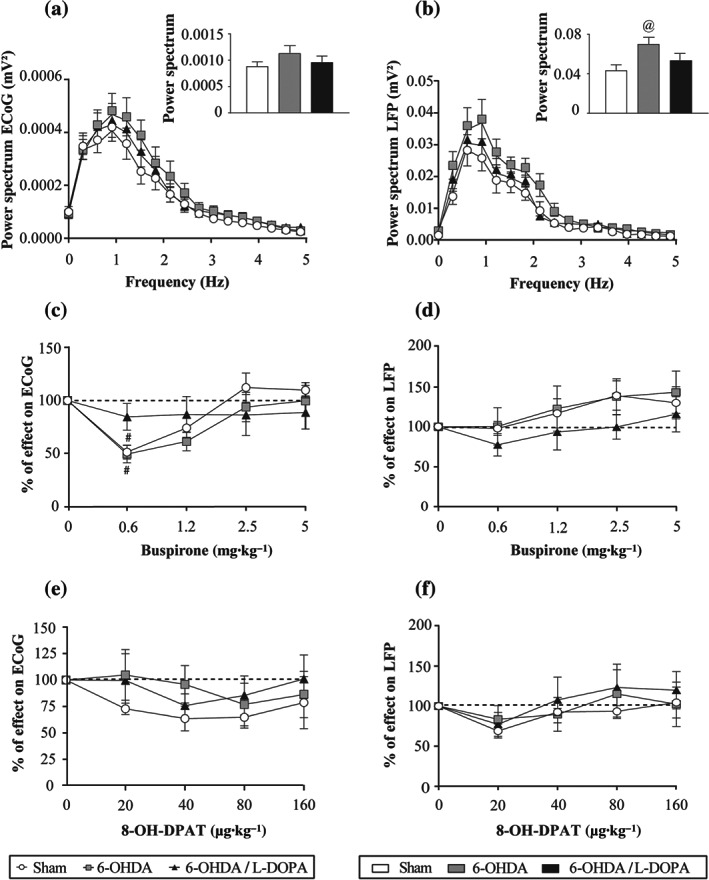
Effect of buspirone on low oscillatory activity and synchronization of SNr neurons and electrocorticogram (ECoG) of the motor cortex. Power spectra (0‐ to 5‐Hz frequency range) and AUC values obtained from the ECoG (a) and SNr‐local field potential (LFP) (b) in the sham (*n* = 19), 6‐OHDA (*n* = 17), and 6‐OHDA/l‐DOPA (*n* = 18) animals. Effect of administration of increasing doses of buspirone (0.6125–5 mg·kg^−1^, i.v.) on the AUC of the power spectrum of the ECoG (c) and SNr‐LFP (d) in the sham (*n* = 8), 6‐OHDA (*n* = 7), and 6‐OHDA/l‐DOPA (*n* = 8) animals. Effect of administration of increasing doses of 8‐OH‐DPAT (20–160 μg·kg^−1^, i.v.) on the AUC of the power spectrum of the ECoG (e) and SNr‐LFP (f) obtained from the three experimental groups, sham (*n* = 5), 6‐OHDA (*n* = 5), and 6‐OHDA/l‐DOPA (*n* = 5). Data are expressed as mean ± SEM. ^@^ *P* < 0.05 versus sham (one‐way ANOVA followed by Bonferroni's post hoc test) and ^#^ *P* < 0.05 versus respective baseline (RM two‐way ANOVA followed by Bonferroni's post hoc test)

**FIGURE 6:**
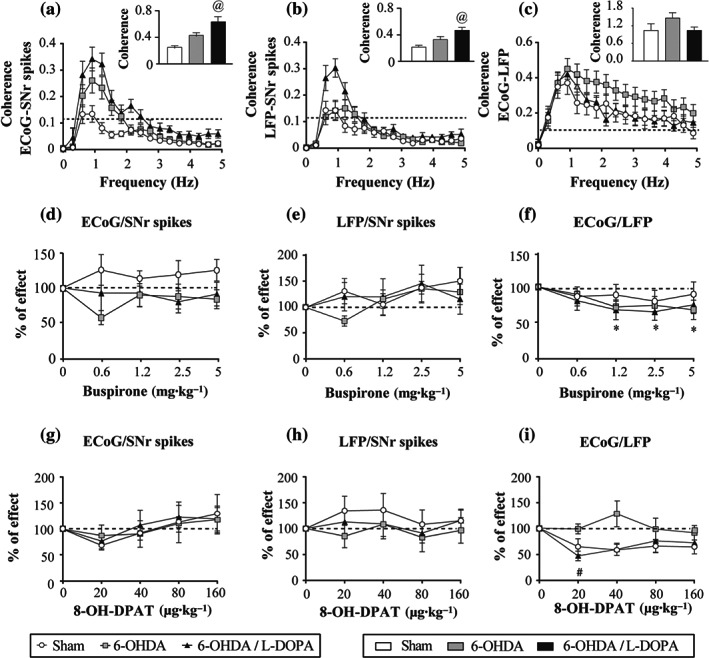
Synchronization between the SNr and electrocorticogram (ECoG) of the motor cortex in low‐frequency range (0.5–5 Hz) was higher in 6‐OHDA‐lesioned groups. Coherence power between the ECoG and SNr spikes simultaneously recorded in the sham (*n* = 19), 6‐OHDA (*n* = 17), and 6‐OHDA/l‐DOPA (*n* = 18) groups. AUC value obtained from coherence between the ECoG and SNr spikes was augmented in 6‐OHDA l‐DOPA group (a). AUC values obtained from SNr‐local field potential (LFP)/SNr spikes coherence curves showed an increase in 6‐OHDA l‐DOPA group (b). AUC values obtained from ECoG/SNr‐LFP coherence curves showed no differences (c). Effect of administration of acute doses of buspirone (0.6125–5 mg·kg^−1^, i.v.) on the synchronization between ECoG and SNr spikes (d), between SNr‐LFP and SNr spikes (e), and between ECoG and SNr‐LFP (f) in the sham (*n* = 8), 6‐OHDA (*n* = 7), and 6‐OHDA/l‐DOPA (*n* = 8) groups. Effect of administration of acute doses of 8‐OH‐DPAT (20–160 μg·kg^−1^, i.v.) on the synchronization between ECoG and SNr spikes (g), between SNr‐LFP and SNr spikes (h), and between ECoG and SNr‐LFP (i) in the sham (*n* = 5), 6‐OHDA (*n* = 5), and 6‐OHDA l‐DOPA (*n* = 5) groups. Data are expressed as mean ± SEM. ^@^ *P* < 0.05 versus sham (one‐way ANOVA followed by Bonferroni's post hoc test), ^*^ *P* < 0.05 versus sham, and ^#^ *P* < 0.05 versus respective baseline (RM two‐way ANOVA followed by Bonferroni's post hoc test)

Simultaneously with the single‐unit recordings of SNr neurons described above, we also recorded SNr‐local field potential and the electrocorticogram. Later, we analysed the power spectrum and the coherence in the 0‐ to 5‐Hz range to determine the parameters of low‐frequency activity and synchronization in the SNr and between this nucleus and the cerebral cortex. In agreement with our previous results (Aristieta et al., 2016), the analysis showed low oscillatory activity in the electrocorticogram and the local field potential with a peak near 1 Hz in all three experimental groups (Figure 5a,b). The compilation of the AUC values of the electrocorticogram power spectrum revealed no significant differences among groups (Figure 5a), whereas the local field potential AUC value was higher in the 6‐OHDA group (Figure 5b). The coherence analysis showed that synchronization between electrocorticogram and SNr spikes and between local field potential and SNr spikes was higher after 6‐OHDA lesion and l‐DOPA treatment, with the AUC value obtained from the coherence curves being significantly larger in the 6‐OHDA and 6‐OHDA/l‐DOPA groups (*P* < 0.05; Figure 6a,b). No difference was observed in the synchronization between electrocorticogram and SNr‐local field potential (Figure 6c).

**FIGURE 7:**
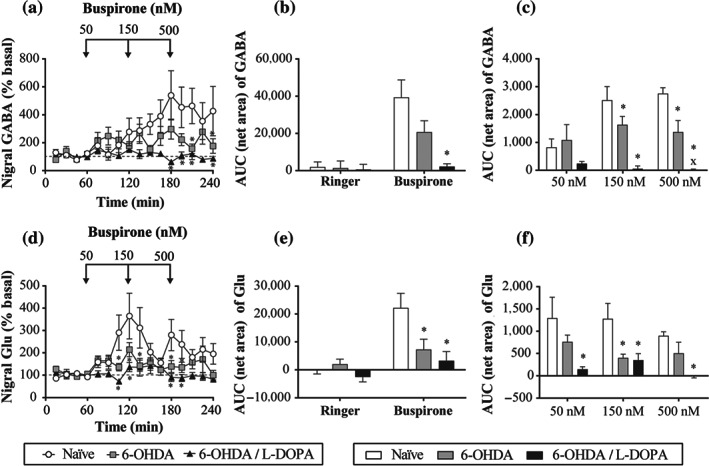
Effect of local perfusion of buspirone (50–500 nM) on nigral amino acid levels of GABA (a–c) and glutamate (Glu) (d–f). Local perfusion of buspirone raised nigral GABA levels in naïve (*n* = 9) and 6‐OHDA (*n* = 11) groups, but with less intensity, and it had no effect in 6‐OHDA/l‐DOPA (*n* = 7) group (a). Buspirone also raised nigral Glu levels in naïve (*n* = 10) and 6‐OHDA (*n* = 9) groups, with less intensity, and it had no effect in 6‐OHDA/l‐DOPA (*n* = 7) group (d). Bar representation (mean ± SEM) of the AUC (60–240 min) (b and e) and AUC 60 min after administration of the respective buspirone concentration dose (c and f). Data are expressed as percentage of basal levels calculated as the mean of the two samples preceding buspirone perfusion. ^*^ *P* < 0.05 versus naïve group and ^x^ *P* < 0.05 versus respective dose of 6‐OHDA group (RM two‐way ANOVA followed by Bonferroni's post hoc test)

Next, to investigate the effect of 5‐HT_1A_ drugs on low oscillatory activity and synchronization between SNr and electrocorticogram, we compared the power spectrum and coherence parameters obtained before and after the systemic administration of increasing doses of buspirone or 8‐OH‐DPAT. Buspirone administration produced a significant effect on electrocorticogram (Figure 5c) and local field potential (Figure 5d), but this effect was only significant for the first electrocorticogram dose in sham and 6‐OHDA groups. On the other hand, buspirone administration did not affect the synchronization between electrocorticogram and SNr spikes (Figure 6d). Nevertheless, buspirone produced a slight but significant effect between local field potential and SNr spikes (*P* < 0.05; Figure 6e). Synchronization between electrocorticogram and local field potential was not affected much by buspirone (Figure 6f).

Finally, we investigated the effect of systemic 8‐OH‐DPAT administration on the oscillatory activity and synchronization. As it is shown in Figure 5e,f, 8‐OH‐DPAT did not cause any change in the AUC of the electrocorticogram and local field potential power spectrum. In addition, analysis of coherence revealed that 8‐OH‐DPAT had a slight effect on AUC of the coherence between electrocorticogram and SNr spikes, but no differences between the three experimental groups were observed (Figure 6g). 8‐OH‐DPAT did not cause any effect on the AUC of the coherence between local field potential and SNr spikes (Figure 6h). In the coherence between electrocorticogram and local field potential, there was a significant reduction in 6‐OHDA/l‐DOPA group with the first dose of 8‐OH‐DPAT, but no effects were observed for subsequent doses (Figure 6i).

### 3.3 Effect of buspirone on amino acid release in the SNr from conscious‐freely moving rats

We next investigated whether buspirone was able to modulate nigral GABA and glutamate levels. Immediately before buspirone perfusion, basal GABA and glutamate levels in the dialysate from the probe implanted in the SNr did not differ among the three experimental groups 7.28 ± 2.65 nM and 94.35 ± 11.99 nM, respectively, in naïve animals (*n* = 11), 8.44 ± 1.89 nM and 110.27 ± 16.01, respectively, in 6‐OHDA animals (*n* = 11), and 8.73 ± 1.58 and 71.87 ± 7.65, respectively, in 6‐OHDA l‐DOPA animals (*n* = 8). Local perfusion of buspirone (50, 150 and 500 nM) raised dialysate GABA levels in naïve and 6‐OHDA groups but had no effect in 6‐OHDA/l‐DOPA animals (Figure 7a). However, the GABA enhancement was significantly lower in the 6‐OHDA group when compared to that in the naïve group (Figure 7b,c). Likewise, buspirone also elevated dialysate glutamate release in naïve and 6‐OHDA groups, but it had no effect in 6‐OHDA/l‐DOPA animals (Figure 7d–f). Again, the effect of buspirone on 6‐OHDA‐lesioned rats was significantly less when compared to the effect on naïve rats (Figure 7d,e,f).

### 3.4 SERT and 5‐HT_1A_ immunostaining in the SNr

**FIGURE 8:**
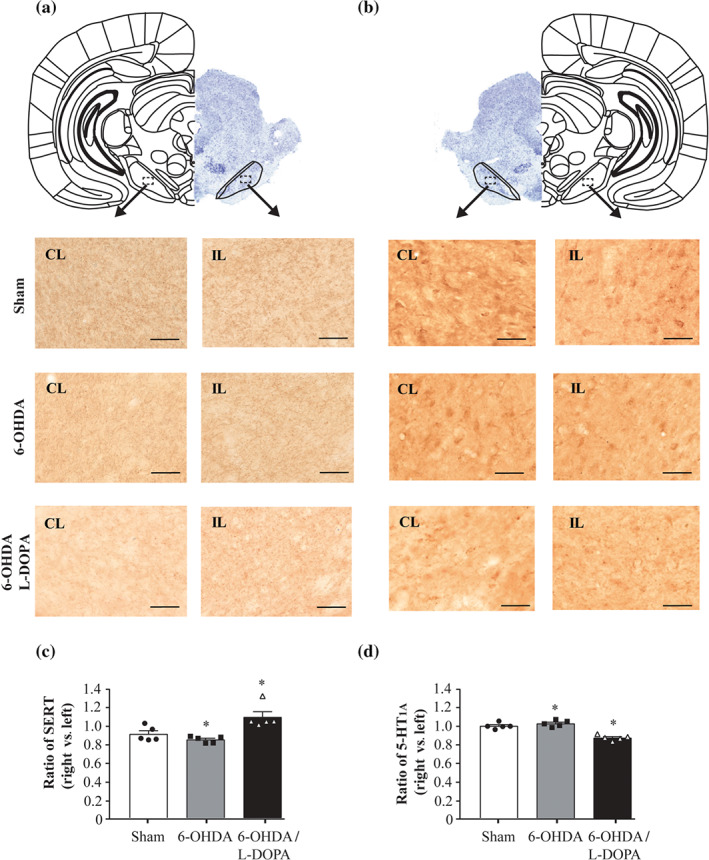
Coronal sections immunoassayed for SERT axon terminals (a) and 5‐HT_1A_ receptors (b) of the SNr. Micrographs at 20× magnifications for the three experimental groups. In the top of the figure, a representative brain coronal section of the SNr (Nissl staining) with delimited sections from ipsilateral (IP) and contralateral (CL) hemispheres. Scale bar = 50 μm. While the IOD of SERT terminals left/right ratio shows a significant increase in SERT terminals (c), 5‐HT_1A_ receptor left/right ratio shows a significant decrease in 6‐OHDA/l‐DOPA group (d). Sham (*n* = 5), 6‐OHDA‐lesioned (*n* = 5), and 6‐OHDA/l‐DOPA (*n* = 5). Each bar represents the mean ± SEM. ^*^ *P* < 0.05 versus sham (one‐way ANOVA followed by Bonferroni's post hoc test)

Published studies concerning 5‐HT innervations and 5‐HT_1A_ receptors in the basal ganglia after 6‐OHDA lesion have shown uniform results (see Miguelez, Morera‐Herreras, Torrecilla, Ruiz‐Ortega, & Ugedo, 2014). Therefore, in order to investigate the mechanism involved in the observed changes produced by buspirone after 6‐OHDA lesion, we evaluated SERT and 5‐HT_1A_ immunoreactivity expression in the SNr of animals in the three experimental groups (sham *n* = 5, 6‐OHDA *n* = 5 and 6‐OHDA/l‐DOPA *n* = 5 animals). The overall integrated optical densitometry for SERT or 5‐HT_1A_ immunoreactivity was unchanged by the 6‐OHDA lesion. By contrast, prolonged l‐DOPA treatment significantly increased SERT (Figure 8c) and significantly decreased 5‐HT_1A_ receptor expression (Figure 8d).

## 4 DISCUSSION

We have used 6‐OHDA‐lesioned rats, a well‐characterized experimental model of Parkinson's disease, to elucidate whether the substantia nigra pars reticulata (SNr) could be a good target to evaluate pharmacological treatments for the motor symptoms associated with Parkinsonism. Buspirone is one of the most effective drugs in reducing motor symptoms and it can reverse certain molecular changes induced by l‐DOPA in parkinsonian rats (Azkona et al., 2014; Dekundy et al., 2007; Eskow, Gupta, Alam, Park, & Bishop, 2007). Here, we show that in anaesthetized rats buspirone (1) reduces the firing rate of SNr neurons when administered directly to the nucleus, (2) decreases bursty activity when it is applied systemically after DA loss but not in control conditions and (3) has no effect on local field potential and synchronization between the cortex and the SNr and within the nucleus. In addition, buspirone modulates GABA and glutamate levels depending on the integrity of DA pathways. Thus, amino acid levels were increased in control conditions, were little affected after DA degeneration and were not modified after 6‐OHDA lesion and l‐DOPA treatment.

### 4.1 Effect of buspirone on SNr neuron activity

Although numerous behavioural studies have shown the efficacy of full or partial 5‐HT_1A_ receptor agonists in decreasing dyskinesias, no study has examined how these drugs may affect the altered output basal ganglia activity in Parkinson's disease and l‐DOPA‐induced dyskinesia. Here, we first show that the firing rate of SNr neurons was reduced when buspirone was administered directly into the nucleus. However, buspirone caused no change in the firing rate of SNr neurons when administered systemically, whereas 8‐OH‐DPAT dose‐dependently reduced it. The effects of local as well as systemic 8‐OH‐DPAT can be attributed to 5‐HT_1A_ receptor activation since they were antagonized by WAY‐100635. Altogether, these results indicate that the reduction of firing rate involves 5‐HT_1A_ receptors located within the SNr and 5‐HT_1A_ receptors located on excitatory and inhibitory neurons that project to the SNr. Thus, the systemic effects of buspirone and 8‐OH‐DPAT depend first on the inhibition of 5‐HT neurons in the raphe nuclei and the activity modulation of the striatum, globus pallidus and the subthalamic nucleus and second on the direct activation of 5‐HT_1A_ receptors in the SNr. In fact, the dorsal raphé nucleus sends efferentes to the SNr (Moukhles et al., 1997) as well as to the other basal nuclei (see Di Matteo et al., 2008).

Differences between the effects of buspirone and 8‐OH‐DPAT likely rely on the fact that 8‐OH‐DPAT is a full agonist at presynaptic and postsynaptic 5‐HT_1A_ receptors (which results in inhibition of the spontaneous firing rate of 5‐HT neurons and 5‐HT release) and reduces inhibitory responses on targeted structures (Blier, Lista, & De Montigny, 1993; Martín‐Ruiz & Ugedo, 2001; Sprouse & Aghajanian, 1988). Conversely, buspirone acts as a full agonist at presynaptic 5‐HT_1A_ receptors inhibiting 5‐HT neurons (VanderMaelen, Matheson, Wilderman, & Patterson, 1986) but as a partial agonist of postsynaptic 5‐HT_1A_ receptors (Cowen, Power, Ware, & Anderson, 1994; Eison & Temple, 1986), where its effect will depend on 5‐HT levels at the synaptic cleft. Notably, following DA depletion and l‐DOPA treatment, the effect of 8‐OH‐DPAT was abolished. In that sense, several studies have shown that dysfunction of 5‐HT_1A_ receptors in rats after DA degeneration decreases or abolishes the effect of 8‐OH‐DPAT on GABAergic interneurons (Hou et al., 2012) and pyramidal neurons (Wang, Zhang, Liu, Wu, Ali, et al., 2009a). However, regarding the effect of systemic 8‐OH‐DPAT administration on dorsal raphé nucleus neurons, a loss in efficacy of inhibition (Wang, Zhang, Liu, Wu, Wang, et al, 2009b) or no changes (Miguelez, Navailles, De Deurwaerdère, & Ugedo, 2016) have been reported. Interestingly, systemic administration of buspirone reduced the number of bursty SNr neurons after DA loss but not in control conditions where 8‐OH‐DPAT has a more pronounced effect. Precisely, an increment in burst activity was the major change we observed in the SNr neurons from the 6‐OHDA and 6‐OHDA/l‐DOPA groups, which is also in line with data from electrophysiological studies in anaesthetized rats, which have reported changes in firing patterns rather than in the firing frequency (Aristieta et al., 2016; Meissner et al., 2006; Tseng et al., 2000). This enhancement in burst activity may be due to changes in synaptic plasticity induced by DA loss (see Lobb, 2014). It seems that bursting activity is more relevant in information‐carrying signals (Chergui, Nomikos, Mathé, Gonon, & Svensson, 1996) and neurotransmitter release rather than single action potentials (Gonon, 1988). Therefore, the decrement in burst activity induced by buspirone will produce less intense GABA releases in agreement with our microdialysis results discussed below.

### 4.2 Effect of buspirone on SNr neuron oscillatory activity and synchronization

Cortical slow‐wave activity induced by urethane anaesthesia favours low‐frequency oscillations in the cortex and the basal ganglia nuclei in rats, including the SNr (Aristieta et al., 2016; Brown, 2006; Clement et al., 2008; Magill, Bolam, & Bevan, 2001). Thus, the later cited studies demonstrated that these electrophysiological properties are altered in 6‐OHDA‐lesioned and l‐DOPA‐treated rats, which show that coupling between the cortex and the SNr increased. In agreement with these findings, we also observed oscillatory activity in the low‐frequency band (0–5 Hz) in the cortex and the SNr, and an increased synchronization between them after DA loss and l‐DOPA treatment. This low‐frequency oscillatory activity and synchronization were not affected by systemic buspirone or 8‐OH‐DPAT administration at the same doses that modified bursty activity. So far, there are no studies regarding the effect of antidyskinetic drugs on this low oscillatory activity, but our previous work found that an acute l‐DOPA challenge had little effect on it (Aristieta et al., 2016). It seems, therefore, that while low‐frequency oscillatory activity and synchronization do not have a relevant role in l‐DOPA‐induced dyskinesia, they may be important in Parkinson's disease. In fact, recent published evidence obtained from 6‐OHDA‐lesioned rats indicates that oscillations in distinct spectral bands are differently affected at every Parkinsonism stage and may be differently modulated by pharmacological treatments. Thus, recordings in awake animals have shown that, apart from decreasing dyskinesia, 5‐HT‐antidyskinetic drugs suppressed 80‐Hz oscillation activity but not the oscillations in other bands (5–10 Hz and 110–140 Hz) (Brys et al., 2018). Similarly, oscillations in the β band (24–36 Hz) were elevated, but those in the θ band (5–8 Hz) were unchanged after the 6‐OHDA lesion. However, θ activity was elevated and correlated with abnormal involuntary movements score in 6‐OHDA‐lesioned rats treated with l‐DOPA and was reduced by eltoprazine, a 5HT_1A/B_ agonist (Wang et al., 2019).

### 4.3 Effect of buspirone on SNr neuron amino acid levels

In this study, using microdialysis approaches we found no significant changes in GABA and glutamate basal levels after DA loss with or without prolonged l‐DOPA treatment. This finding is consistent with those of other studies performed in freely moving 6‐OHDA‐lesioned rats (Bianchi et al., 2003; Mela et al., 2007). However, we found that the enhancement in GABA and glutamate release induced by locally perfused buspirone in control animals was significantly reduced after 6‐OHDA lesion and almost abolished after prolonged l‐DOPA treatment. The sources of GABA in the SNr include the striatal and globus pallidus terminals, and collaterals of GABAergic neurons of the SNr, while the subthalamic nucleus provides the major glutamatergic input to the SNr (Kita & Kitai, 1987). In control conditions, GABA and glutamate release in the SNr may be regulated by 5‐HT_1A_ receptors localized on 5‐HT terminals coming from the raphé nuclei, striatal, globus pallidus and subthalamic terminals and nigral GABAergic neurons. The fact that the inhibitory effect of buspirone on SNr neuron activity was unchanged after 6‐OHDA lesion rules out the possibility that changes in 5‐HT_1A_ receptors on GABAergic neurons are responsible for the reduction of GABA released. However, this may reflect altered responsiveness from afferent terminals as shown for nigral GABA release following striatal kainate application and for glutamate release following local application of K^+^ in 6‐OHDA‐lesioned rats (Bianchi et al., 2003). Furthermore, buspirone, besides being a 5‐HT_1A_ partial agonist also has D_2_ and D_3_/D_4_ receptor antagonist activity (Bergman et al., 2013). These receptors, whose activity is modulated by the 6‐OHDA lesion (Avalos‐Fuentes et al., 2013), are present in the pallidonigral and striatonigral terminals, modulate GABA release (Acosta‐García et al., 2009; Avalos‐Fuentes et al., 2015) and are involved in the antidyskinetic effect of buspirone (Azkona et al., 2014; Shin et al., 2014). Therefore, changes in GABA and glutamate release induced by buspirone may involve modulation of several receptors apart from 5‐HT_1A_ receptor. In fact, in the present study, we only found a decrease in optical density of 5‐HT_1A_ receptors in the SNr from 6‐OHDA‐lesioned rats with prolonged l‐DOPA treatment where buspirone effect on amino acid release was negligible. However, no changes were observed after DA degeneration, while buspirone effect was reduced compared to that in control conditions.

### 4.4 SERT and 5‐HT_1A_ receptor expression in the SNr from 6‐OHDA‐lesioned rats

Published studies regarding SERT and 5‐HT_1A_ receptor changes in Parkinson's disease have provided contradictory results showing increased, decreased or unmodified expression of these proteins both in humans and in animal models. This discrepancy may be due to the stage of the disease, the treatment, or the experimental model used (for review, see Vegas‐Suarez et al., 2019). To better explain our electrophysiological and microdialysis results, we performed immunohistochemical detection of SERT and 5‐HT_1A_ in the same experimental model. Results show that SNr expression of SERT is increased and this observation agrees with the findings of a study involving the striatum of hemiparkinsonian rats (Rylander et al., 2010). The 5‐HT_1A_ receptor density was decreased in that case in disagreement with published results showing no striatal changes (Riahi et al., 2012). However, although in both cases the change is significant, it is only around a 10% of the control value and was only present in 6‐OHDA‐lesioned animals that had undergone prolonged treatment of l‐DOPA. It could be speculated that changes in 5‐HT_1A_ receptors take place on terminals rather than on SNr neurons, which could explain why local buspirone administration has no effect. On the other hand, these results could help to understand the lack of the effect of buspirone on amino acid release in 6‐OHDA‐lesioned and prolonged l‐DOPA‐treated animals but not the decreased effect of buspirone on 6‐OHDA‐lesioned rats. Therefore, it is hard to consider that changes in density are responsible of the altered response to buspirone observed after 6‐OHDA lesion and complex mechanisms underlying the decreasing receptor sensitivity are probably involved.

### 4.5 Experimental considerations

This study was performed only in male rats to avoid gender influence in the results, since electrophysiological differences between male and female have been observed in Parkinson's disease patients (see review Picillo et al., 2017). In this regard, recordings in the STN have shown sex‐related differences in spike parameters and high‐γ band oscillations activity (Marceglia et al., 2006; Mrakic‐Sposta et al., 2008). The development of l‐DOPA‐induced dyskinesia and the response to deep brain stimulation also differ between female and male subjects (Accolla et al., 2007; Hassin‐Baer et al., 2011). In this line, previous work from our laboratory confirmed gender differences in the STN neurons from 6‐OHDA‐lesioned rats with and without l‐DOPA prolonged treatment (Sagarduy et al., 2016). A limitation of the present study is that recordings were performed in anaesthetized animals, and it may be argued that this does not accurately represent clinical situations. However, we have recently characterized the electrophysiological parameters in this model of DA loss and demonstrated a relationship between the observation of abnormal involuntary movements and recording parameters under anaesthesia. Thus, we chose to use buspirone in this study because we knew from previous studies that a single dose of buspirone applied before l‐DOPA was able to reduce abnormal involuntary movements and certain molecular changes observed in 6‐OHDA‐lesioned and l‐DOPA‐treated rats (Azkona et al., 2014; Sagarduy et al., 2016).

## 5 CONCLUSION

The relevance of the SNr in Parkinson's disease and l‐DOPA‐induced dyskinesia is evidenced by aberrant changes of nigral neuron activity observed after DA depletion and l‐DOPA treatment in animal models, as well as the symptomatic efficacy of therapeutic interventions that normalize nigral activity in both animal models and patients with Parkinson's disease. Our results show that buspirone modulates amino acid release and neuronal activity, mainly burst activity, in the SNr of the rat. However, these effects are modulated by DA loss and long‐term l‐DOPA treatment. These novel findings indicate that the regulation of burst activity in the SNr induced by DA loss may be a good target to test new drugs for the treatments of Parkinson's disease and dyskinetic patients and point out the importance of using experimental models of Parkinson's disease and l‐DOPA‐induced dyskinesia when investigating the potential therapeutic effect of new drugs.

## AUTHOR CONTRIBUTIONS

L.U., C.M. and M.M. conceived the study, designed the experiments, and drafted the final version of the manuscript. S.V.S. performed all the experiments, carried out data quantification and analysis, prepared the figures, and contributed to the first draft. C.A.P. carried out some microdialysis experiments. C.R. and H.B. contributed in the immunostaining experiment design. C.M. revised the analysis of data. S.V.S., L.U., J.V.L., C.M., C.A.P. and M.M. interpreted the results. All authors reviewed and edited the manuscript.

## CONFLICT OF INTEREST

The authors declare no conflicts of interest.

## DECLARATION OF TRANSPARENCY AND SCIENTIFIC RIGOUR

This Declaration acknowledges that this paper adheres to the principles for transparent reporting and scientific rigour of preclinical research as stated in the *BJP* guidelines for Design & Analysis, Immunoblotting and Immunochemistry, and Animal Experimentation, and as recommended by funding agencies, publishers, and other organizations engaged with supporting research.

## Supporting information

Figure S1. Validation of 6‐OHDA lesion and L‐DOPA induced abnormal involuntary movements. Evolution of dyskinesia scores showing (A) the time course of AIM scores for axial, limb and orolingual ratings and (B) locomotive score, on the last session after L‐DOPA chronic treatment. Results are expressed as means ± S.E.M. Coronal sections (C) from sham group and 6‐OHDA group. Note the lack of TH immunoreactivity in the striatum and SN. Scale bar = 1 mm.

Figure S2. Effect of buspirone on SNr spike trains and oscillatory activity of the SNr and the cortex. On the left, a recording track containing SNr spikes, SNr‐LFP and ECoG from the basal neuron‐firing pattern in the sham, 6‐OHDA and 6‐OHDA group. On the right, a recording track containing SNr spikes, SNr‐LFP and ECoG from the same neurons after buspirone administration (2.5 mg/kg, i.v.). Note that buspirone did not alter burst‐exhibiting pattern in sham group while buspirone reduce it in 6‐OHDA and 6‐OHDA L‐DOPA groups.

Table S1. Statistical Details

Table S2. Effect of buspirone on firing properties of SNr neurons.

Table S3. Effect of 8‐OH‐DPAT on firing properties of SNr neurons.

## References

[bph15145-bib-0001] Accolla, E. , Caputo, E. , Cogiamanian, F. , Tamma, F. , Mrakic‐Sposta, S. , Marceglia, S. , … Priori, A. (2007). Gender differences in patients with Parkinson's disease treated with subthalamic deep brain stimulation. Movement Disorders, 22(8), 1150–1156. 10.1002/mds.21520 17469208

[bph15145-bib-0002] Acosta‐García, J. , Hernández‐Chan, N. , Paz‐Bermúdez, F. , Sierra, A. , Erlij, D. , Aceves, J. , & Florán, B. (2009). D4 and D1 dopamine receptors modulate [3H]GABA release in the substantia nigra pars reticulata of the rat. Neuropharmacology, 57(7–8), 725–730. 10.1016/j.neuropharm.2009.08.010 19715708

[bph15145-bib-0003] Alexander, S. P. H. , Roberts, R. E. , Broughton, B. R. S. , Sobey, C. G. , George, C. H. , Stanford, S. C. , … Ahluwalia, A. (2018). Goals and practicalities of immunoblotting and immunohistochemistry: A guide for submission to the British Journal of Pharmacology. British Journal of Pharmacology. John Wiley and Sons Inc, 175, 407–411. 10.1111/bph.14112 PMC5773976 29350411

[bph15145-bib-0004] Arcuri, L. , Novello, S. , Frassineti, M. , Mercatelli, D. , Pisanò, C. A. , Morella, I. , … Morari, M. (2018). Anti‐Parkinsonian and anti‐dyskinetic profiles of two novel potent and selective nociceptin/orphanin FQ receptor agonists. British Journal of Pharmacology, 175(5), 782–796. 10.1111/bph.14123 29232769 PMC5811622

[bph15145-bib-0005] Aristieta, A. , Azkona, G. , Sagarduy, A. , Miguelez, C. , Ruiz‐Ortega, J. Á. , Sanchez‐Pernaute, R. , & Ugedo, L. (2012). The role of the subthalamic nucleus in L‐DOPA induced dyskinesia in 6‐hydroxydopamine lesioned rats. PLoS ONE, 7(8), e42652,1–14. 10.1371/journal.pone.0042652 PMC3412805 22880070

[bph15145-bib-0006] Aristieta, A. , Ruiz‐Ortega, J. A. , Miguelez, C. , Morera‐Herreras, T. , & Ugedo, L. (2016). Chronic L‐DOPA administration increases the firing rate but does not reverse enhanced slow frequency oscillatory activity and synchronization in substantia nigra pars reticulata neurons from 6‐hydroxydopamine‐lesioned rats. Neurobiology of Disease, 89, 88–100. 10.1016/j.nbd.2016.02.003 26852950

[bph15145-bib-0007] Aristieta, A. , Ruiz‐Ortega, J. A. , Morera‐Herreras, T. , Miguelez, C. , & Ugedo, L. (2019). Acute L‐DOPA administration reverses changes in firing pattern and low frequency oscillatory activity in the entopeduncular nucleus from long term L‐DOPA treated 6‐OHDA‐lesioned rats. Experimental Neurology, 322, 113036,1–14. 10.1016/j.expneurol.2019.113036 31425688

[bph15145-bib-0008] Avalos‐Fuentes, A. , Albarrán‐Bravo, S. , Loya‐Lopéz, S. , Cortés, H. , Recillas‐Morales, S. , Magaña, J. J. , … Florán, B. (2015). Dopaminergic denervation switches dopamine D3 receptor signaling and disrupts its Ca2+ dependent modulation by CaMKII and calmodulin in striatonigral projections of the rat. Neurobiology of Disease, 74, 336–346. 10.1016/j.nbd.2014.12.008 25517101

[bph15145-bib-0009] Avalos‐Fuentes, A. , Loya‐López, S. , Flores‐Pérez, A. , Recillas‐Morales, S. , Cortés, H. , Paz‐Bermúdez, F. , … Florán, B. (2013). Presynaptic CaMKIIα modulates dopamine D3 receptor activation in striatonigral terminals of the rat brain in a Ca2+ dependent manner. Neuropharmacology, 71, 273–281. 10.1016/j.neuropharm.2013.04.010 23602989

[bph15145-bib-0010] Azkona, G. , Sagarduy, A. , Aristieta, A. , Vazquez, N. , Zubillaga, V. , Ruíz‐Ortega, J. A. , … Sánchez‐Pernaute, R. (2014). Buspirone anti‐dyskinetic effect is correlated with temporal normalization of dysregulated striatal DRD1 signalling in l‐DOPA‐treated rats. Neuropharmacology, 79, 726–737. 10.1016/j.neuropharm.2013.11.024 24333147

[bph15145-bib-0011] Bergman, J. , Roof, R. A. , Furman, C. A. , Conroy, J. L. , Mello, N. K. , Sibley, D. R. , & Skolnick, P. (2013). Modification of cocaine self‐administration by buspirone (buspar®): Potential involvement of D_3_ and D_4_ dopamine receptors. International Journal of Neuropsychopharmacology, 16(2), 445–458. 10.1017/S1461145712000661 22827916 PMC5100812

[bph15145-bib-0012] Bianchi, L. , Ballini, C. , Colivicchi, M. A. , Della Corte, L. , Giovannini, M. G. , & Pepeu, G. (2003). Investigation on acetylcholine, aspartate, glutamate and GABA extracellular levels from ventral hippocampus during repeated exploratory activity in the rat. Neurochemical Research, 28(3–4), 565–573. 10.1023/a:1022881625378 12675146

[bph15145-bib-0013] Bibbiani, F. , Oh, J. D. , & Chase, T. N. (2001). Serotonin 5‐HT1A agonist improves motor complications in rodent and primate parkinsonian models. Neurology, 57(10), 1829–1834. 10.1212/WNL.57.10.1829 11723272

[bph15145-bib-0014] Bido, S. , Marti, M. , & Morari, M. (2011). Amantadine attenuates levodopa‐induced dyskinesia in mice and rats preventing the accompanying rise in nigral GABA levels. Journal of Neurochemistry, 118(6), 1043–1055. 10.1111/j.1471-4159.2011.07376.x 21740438

[bph15145-bib-0015] Bishop, C. , George, J. A. , Buchta, W. , Goldenberg, A. A. , Mohamed, M. , Dickinson, S. O. , … Eskow Jaunarajs, K. L. (2012). Serotonin transporter inhibition attenuates l‐DOPA‐induced dyskinesia without compromising l‐DOPA efficacy in hemi‐parkinsonian rats. The European Journal of Neuroscience, 36(6), 2839–2848. 10.1111/j.1460-9568.2012.08202.x 22762478 PMC3445783

[bph15145-bib-0016] Blier, P. , Lista, A. , & De Montigny, C. (1993). Differential properties of pre‐ and postsynaptic 5‐hydroxytryptamine1A receptors in the dorsal raphe and hippocampus: I. Effect of spiperone. The Journal of Pharmacology and Experimental Therapeutics, 265(1), 7–15. Retrieved from. http://www.ncbi.nlm.nih.gov/pubmed/8474032 8474032

[bph15145-bib-0017] Brown, P. (2006). Bad oscillations in Parkinson's disease In Parkinson's disease and related disorders (pp. 27–30). Vienna: Springer Vienna 10.1007/978-3-211-45295-0_6

[bph15145-bib-0018] Brys, I. , Halje, P. , Scheffer‐Teixeira, R. , Varney, M. , Newman‐Tancredi, A. , & Petersson, P. (2018). Neurophysiological effects in cortico‐basal ganglia‐thalamic circuits of antidyskinetic treatment with 5‐HT 1A receptor biased agonists. Experimental Neurology, 302, 155–168. 10.1016/j.expneurol.2018.01.010 29339052

[bph15145-bib-0019] Caretti, V. , Stoffers, D. , Winogrodzka, A. , Isaias, I.‐U. , Costantino, G. , Pezzoli, G. , … Booij, J. (2008). Loss of thalamic serotonin transporters in early drug‐naïve Parkinson's disease patients is associated with tremor: An [(123)I]β‐CIT SPECT study. Journal of Neural Transmission (Vienna, Austria: 1996), 115(5), 721–729. 10.1007/s00702-007-0015-2 PMC2440940 18335163

[bph15145-bib-0020] Cenci, M. A. , & Lundblad, M. (2007). Ratings of L‐DOPA‐induced dyskinesia in the unilateral 6‐OHDA lesion model of Parkinson's disease in rats and mice In Current protocols in neuroscience (Vol. 41). John Wiley & Sons, Inc. 10.1002/0471142301.ns0925s41 18428668

[bph15145-bib-0021] Chergui, K. , Nomikos, G. G. , Mathé, J. M. , Gonon, F. , & Svensson, T. H. (1996). Burst stimulation of the medial forebrain bundle selectively increases Fos‐like immunoreactivity in the limbic forebrain of the rat. Neuroscience, 72(1), 141–156. 10.1016/0306-4522(95)00513-7 8730713

[bph15145-bib-0022] Clement, E. A. , Richard, A. , Thwaites, M. , Ailon, J. , Peters, S. , & Dickson, C. T. (2008). Cyclic and sleep‐like spontaneous alternations of brain state under urethane anaesthesia. PLoS ONE, 3(4), e2004,1–15. 10.1371/journal.pone.0002004 18414674 PMC2289875

[bph15145-bib-0023] Collomb‐Clerc, A. , & Welter, M. L. (2015). Effects of deep brain stimulation on balance and gait in patients with Parkinson's disease: A systematic neurophysiological review. Neurophysiologie CliniqueElsevier Masson SAS, 45, 371–388. 10.1016/j.neucli.2015.07.001 26319759

[bph15145-bib-0024] Cowen, P. J. , Power, A. C. , Ware, C. J. , & Anderson, I. M. (1994). 5‐HT1A receptor sensitivity in major depression. A neuroendocrine study with buspirone. The British Journal of Psychiatry: The Journal of Mental Science, 164(3), 372–379. 10.1192/bjp.164.3.372 8199791

[bph15145-bib-0025] Curtis, M. J. , Alexander, S. , Cirino, G. , Docherty, J. R. , George, C. H. , Giembycz, M. A. , … Ahluwalia, A. (2018). Experimental design and analysis and their reporting II: Updated and simplified guidance for authors and peer reviewers. British Journal of Pharmacology, 175(7), 987–993. 10.1111/bph.14153 29520785 PMC5843711

[bph15145-bib-0026] Dekundy, A. , Lundblad, M. , Danysz, W. , & Cenci, M. A. (2007). Modulation of l‐DOPA‐induced abnormal involuntary movements by clinically tested compounds: Further validation of the rat dyskinesia model. Behavioural Brain Research, 179(1), 76–89. 10.1016/j.bbr.2007.01.013 17306893

[bph15145-bib-0027] Di Matteo, V. , Pierucci, M. , Esposito, E. , Crescimanno, G. , Benigno, A. , & Di Giovanni, G. (2008). Serotonin modulation of the basal ganglia circuitry: Therapeutic implication for Parkinson's disease and other motor disorders. Progress in Brain Research, 172, 423–463. 10.1016/S0079-6123(08)00921-7 18772045

[bph15145-bib-0028] Doder, M. , Rabiner, E. A. , Turjanski, N. , Lees, A. J. , & Brooks, D. J. (2003). Tremor in Parkinson's disease and serotonergic dysfunction: An 11C‐WAY 100635 PET study. Neurology, 60(4), 601–605. 10.1212/01.WNL.0000031424.51127.2B 12601099

[bph15145-bib-0029] Eison, A. S. , & Temple, D. L. (1986). Buspirone: Review of its pharmacology and current perspectives on its mechanism of action. The American Journal of Medicine, 80(3B), 1–9. 10.1016/0002-9343(86)90325-6 2870639

[bph15145-bib-0030] Eskow, K. L. , Gupta, V. , Alam, S. , Park, J. Y. , & Bishop, C. (2007). The partial 5‐HT1A agonist buspirone reduces the expression and development of l‐DOPA‐induced dyskinesia in rats and improves l‐DOPA efficacy. Pharmacology Biochemistry and Behavior, 87(3), 306–314. 10.1016/J.PBB.2007.05.002 17553556

[bph15145-bib-0031] Gonon, F. G. (1988). Nonlinear relationship between impulse flow and dopamine released by rat midbrain dopaminergic neurons as studied by in vivo electrochemistry. Neuroscience, 24(1), 19–28. 10.1016/0306-4522(88)90307-7 3368048

[bph15145-bib-0032] Grégoire, L. , Samadi, P. , Graham, J. , Bédard, P. J. , Bartoszyk, G. D. , & Di Paolo, T. (2009). Low doses of sarizotan reduce dyskinesias and maintain antiparkinsonian efficacy of L‐Dopa in parkinsonian monkeys. Parkinsonism & Related Disorders, 15(6), 445–452. 10.1016/j.parkreldis.2008.11.001 19196540

[bph15145-bib-0033] Hamadjida, A. , Nuara, S. G. , Bédard, D. , Gaudette, F. , Beaudry, F. , Gourdon, J. C. , & Huot, P. (2018). The highly selective 5‐HT2A antagonist EMD‐281,014 reduces dyskinesia and psychosis in the l‐DOPA‐treated parkinsonian marmoset. Neuropharmacology, 139, 61–67. 10.1016/j.neuropharm.2018.06.038 29969592

[bph15145-bib-0034] Harding, S. D. , Sharman, J. L. , Faccenda, E. , Southan, C. , Pawson, A. J. , Ireland, S. , … Davies, J. A. (2018). The IUPHAR/BPS Guide to PHARMACOLOGY in 2018: Updates and expansion to encompass the new guide to IMMUNOPHARMACOLOGY. Nucleic Acids Research, 46(D1), D1091–D1106. 10.1093/nar/gkx1121 29149325 PMC5753190

[bph15145-bib-0035] Hassin‐Baer, S. , Molchadski, I. , Cohen, O. S. , Nitzan, Z. , Efrati, L. , Tunkel, O. , … Korczyn, A. D. (2011). Gender effect on time to levodopa‐induced dyskinesias. Journal of Neurology, 258(11), 2048–2053. 10.1007/s00415-011-6067-0 21533825

[bph15145-bib-0036] Hidding, U. , Gulberti, A. , Pflug, C. , Choe, C. , Horn, A. , Prilop, L. , … Pötter‐Nerger, M. (2019). Modulation of specific components of sleep disturbances by simultaneous subthalamic and nigral stimulation in Parkinson's disease. Parkinsonism & Related Disorders, 62, 141–147. 10.1016/j.parkreldis.2018.12.026 30616868

[bph15145-bib-0037] Hou, C. , Xue, L. , Feng, J. , Zhang, L. , Wang, Y. , Chen, L. , … Liu, J. (2012). Unilateral lesion of the nigrostriatal pathway decreases the response of GABA interneurons in the dorsal raphe nucleus to 5‐HT_1A_ receptor stimulation in the rat. Neurochemistry International, 61(8), 1344–1356. 10.1016/j.neuint.2012.09.012 23032407

[bph15145-bib-0038] Keifman, E. , Ruiz‐DeDiego, I. , Pafundo, D. E. , Paz, R. M. , Solís, O. , Murer, M. G. , & Moratalla, R. (2019). Optostimulation of striatonigral terminals in substantia nigra induces dyskinesia that increases after L‐DOPA in a mouse model of Parkinson's disease. British Journal of Pharmacology, 176(13), 2146–2161. 10.1111/bph.14663 30895594 PMC6555865

[bph15145-bib-0039] Kilkenny, C. , Browne, W. , Cuthill, I. C. , Emerson, M. , & Altman, D. G. (2010). Animal research: Reporting in vivo experiments: The ARRIVE guidelines. British Journal of Pharmacology, 160, 1577–1579. 10.1111/j.1476-5381.2010.00872.x 20649561 PMC2936830

[bph15145-bib-0040] Kish, S. J. , Shannak, K. , & Hornykiewicz, O. (1988). Uneven pattern of dopamine loss in the striatum of patients with idiopathic Parkinson's disease. New England Journal of Medicine, 318(14), 876–880. 10.1056/NEJM198804073181402 3352672

[bph15145-bib-0041] Kita, H. , & Kitai, S. T. (1987). Efferent projections of the subthalamic nucleus in the rat: Light and electron microscopic analysis with the PHA‐L method. The Journal of Comparative Neurology, 260(3), 435–452. 10.1002/cne.902600309 2439552

[bph15145-bib-0042] Lindgren, H. S. , Andersson, D. R. , Lagerkvist, S. , Nissbrandt, H. , & Cenci, M. A. (2010). l‐DOPA‐induced dopamine efflux in the striatum and the substantia nigra in a rat model of Parkinson's disease: Temporal and quantitative relationship to the expression of dyskinesia. Journal of Neurochemistry, 112(6), 1465–1476. 10.1111/j.1471-4159.2009.06556.x 20050978

[bph15145-bib-0043] Loane, C. , Wu, K. , Bain, P. , Brooks, D. J. , Piccini, P. , & Politis, M. (2013). Serotonergic loss in motor circuitries correlates with severity of action‐postural tremor in PD. Neurology, 80(20), 1850–1855. 10.1212/WNL.0b013e318292a31d 23596065 PMC3908354

[bph15145-bib-0044] Lobb, C. J. (2014). Abnormal bursting as a pathophysiological mechanism in Parkinson's disease. Basal Ganglia., 3, 187–195. 10.1016/j.baga.2013.11.002 24729952 PMC3979569

[bph15145-bib-0045] Magill, P. J. , Bolam, J. P. , & Bevan, M. D. (2001). Dopamine regulates the impact of the cerebral cortex on the subthalamic nucleus‐globus pallidus network. Neuroscience, 106(2), 313–330. 10.1016/S0306-4522(01)00281-0 11566503

[bph15145-bib-0046] Marceglia, S. , Mrakic‐Sposta, S. , Foffani, G. , Cogiamanian, F. , Caputo, E. , Egidi, M. , … Priori, A. (2006). Gender‐related differences in the human subthalamic area: A local field potential study. European Journal of Neuroscience, 24(11), 3213–3222. 10.1111/j.1460-9568.2006.05208.x 17156382

[bph15145-bib-0047] Martín‐Ruiz, R. , & Ugedo, L. (2001). Electrophysiological evidence for postsynaptic 5‐HT_1A_ receptor control of dorsal raphe 5‐HT neurones. Neuropharmacology, 41(1), 72–78. 10.1016/s0028-3908(01)00050-8 11445187

[bph15145-bib-0048] Meissner, W. , Ravenscroft, P. , Reese, R. , Harnack, D. , Morgenstern, R. , Kupsch, A. , … Boraud, T. (2006). Increased slow oscillatory activity in substantia nigra pars reticulata triggers abnormal involuntary movements in the 6‐OHDA‐lesioned rat in the presence of excessive extracelullar striatal dopamine. Neurobiology of Disease, 22(3), 586–598. 10.1016/j.nbd.2006.01.009 16531050

[bph15145-bib-0049] Mela, F. , Marti, M. , Dekundy, A. , Danysz, W. , Morari, M. , & Cenci, M. A. (2007). Antagonism of metabotropic glutamate receptor type 5 attenuates L‐DOPA‐induced dyskinesia and its molecular and neurochemical correlates in a rat model of Parkinson's disease. Journal of Neurochemistry, 101(2), 483–497. 10.1111/j.1471-4159.2007.04456.x 17359492

[bph15145-bib-0050] Miguelez, C. , Aristieta, A. , Cenci, M. A. , & Ugedo, L. (2011). The locus coeruleus is directly implicated in L‐DOPA‐induced dyskinesia in parkinsonian rats: An electrophysiological and behavioural study. PLoS ONE, 6(9), e24679, 1–12. 10.1371/journal.pone.0024679 21931808 PMC3170382

[bph15145-bib-0051] Miguelez, C. , Navailles, S. , De Deurwaerdère, P. , & Ugedo, L. (2016). The acute and long‐term L‐DOPA effects are independent from changes in the activity of dorsal raphe serotonergic neurons in 6‐OHDA lesioned rats. British Journal of Pharmacology, 173, 2135–2146. 10.1111/bph.13447 26805402 PMC4908202

[bph15145-bib-0052] Miguelez, C. , Benazzouz, A. , Ugedo, L. , & De Deurwaerdère, P. (2017). Impairment of serotonergic transmission by the antiparkinsonian drug L‐DOPA: Mechanisms and clinical implications. Frontiers in Cellular Neuroscience, 11(274), 1–7. 10.3389/fncel.2017.00274 28955204 PMC5600927

[bph15145-bib-0053] Miguelez, C. , Morera‐Herreras, T. , Torrecilla, M. , Ruiz‐Ortega, J. A. , & Ugedo, L. (2014). Interaction between the 5‐HT system and the basal ganglia: Functional implication and therapeutic perspective in Parkinson's disease. Frontiers in Neural Circuits, 8(21), 1–8. 10.3389/fncir.2014.00021 24672433 PMC3955837

[bph15145-bib-0054] Morari, M. , O'Connor, W. T. , Ungerstedt, U. , Bianchi, C. , & Fuxe, K. (1996). Functional neuroanatomy of the nigrostriatal and striatonigral pathways as studied with dual probe microdialysis in the awake rat—II. Evidence for striatal *N*‐methyl‐d‐aspartate receptor regulation of striatonigral gabaergic transmission and motor function. Neuroscience, 72(1), 89–97. 10.1016/0306-4522(95)00556-0 8730708

[bph15145-bib-0055] Moukhles, H. , Bosler, O. , Bolam, J. P. , Vallée, A. , Umbriaco, D. , Geffard, M. , & Doucet, G. (1997). Quantitative and morphometric data indicate precise cellular interactions between serotonin terminals and postsynaptic targets in rat substantia nigra. Neuroscience, 76(4), 1159–1171. 10.1016/S0306-4522(96)00452-6 9027876

[bph15145-bib-0056] Mrakic‐Sposta, S. , Marceglia, S. , Egidi, M. , Carrabba, G. , Rampini, P. , Locatelli, M. , … Priori, A. (2008). Extracellular spike microrecordings from the subthalamic area in Parkinson's disease. Journal of Clinical Neuroscience, 15(5), 559–567. 10.1016/j.jocn.2007.02.091 18378458

[bph15145-bib-0057] Olanow, C. W. (2008). Levodopa/dopamine replacement strategies in Parkinson's disease—Future directions. Movement Disorders, 23(S3), S613–S622. 10.1002/mds.22061 18781663

[bph15145-bib-0058] Paxinos, G. , & Watson, C. (1997). The rat brain, in stereotaxic coordinates. Academic Press. 10.1016/0165-0270(80)90021-7 6110810

[bph15145-bib-0059] Perez‐Lloret, S. , & Rascol, O. (2018). Efficacy and safety of amantadine for the treatment of l‐DOPA‐induced dyskinesia. Journal of Neural Transmission, 125, 1237–1250. 10.1007/s00702-018-1869-1 29511826

[bph15145-bib-0060] Picillo, M. , Nicoletti, A. , Fetoni, V. , Garavaglia, B. , Barone, P. , & Pellecchia, M. T. (2017). The relevance of gender in Parkinson's disease: A review. Journal of NeurologyDr. Dietrich Steinkopff Verlag GmbH and co. KG, 264, 1583–1607. 10.1007/s00415-016-8384-9 28054129

[bph15145-bib-0061] Politis, M. , Wu, K. , Loane, C. , Brooks, D. J. , Kiferle, L. , Turkheimer, F. E. , … Piccini, P. (2014). Serotonergic mechanisms responsible for levodopa‐induced dyskinesias in Parkinson's disease patients. The Journal of Clinical Investigation, 124(3), 1340–1349. 10.1172/JCI71640 24531549 PMC3934188

[bph15145-bib-0062] Qamhawi, Z. , Towey, D. , Shah, B. , Pagano, G. , Seibyl, J. , Marek, K. , … Pavese, N. (2015). Clinical correlates of raphe serotonergic dysfunction in early Parkinson's disease. Brain, 138(10), 2964–2973. 10.1093/brain/awv215 26209314

[bph15145-bib-0063] Riahi, G. , Morissette, M. , Lévesque, D. , Rouillard, C. , Samadi, P. , Parent, M. , & Paolo, T. D. (2012). Effect of chronic l‐DOPA treatment on 5‐HT1A receptors in parkinsonian monkey brain. Neurochemistry International, 61(7), 1160–1171. 10.1016/j.neuint.2012.08.009 22940695

[bph15145-bib-0064] Rosenberg, J. R. , Amjad, A. M. , Breeze, P. , Brillinger, D. R. , & Halliday, D. M. (1989). The Fourier approach to the identification of functional coupling between neuronal spike trains. Progress in Biophysics and Molecular Biology, 53(1), 1–31. 10.1016/0079-6107(89)90004-7 2682781

[bph15145-bib-0065] Rylander, D. , Parent, M. , O'Sullivan, S. S. , Dovero, S. , Lees, A. J. , Bezard, E. , … Cenci, M. A. (2010). Maladaptive plasticity of serotonin axon terminals in levodopa‐induced dyskinesia. Annals of Neurology, 68(5), 619–628. 10.1002/ana.22097 20882603

[bph15145-bib-0066] Sagarduy, A. , Llorente, J. , Miguelez, C. , Morera‐Herreras, T. , Ruiz‐Ortega, J. A. , & Ugedo, L. (2016). Buspirone requires the intact nigrostriatal pathway to reduce the activity of the subthalamic nucleus via 5‐HT1A receptors. Experimental Neurology, 277, 35–45. 10.1016/j.expneurol.2015.12.005 26687972

[bph15145-bib-0067] Shin, E. , Lisci, C. , Tronci, E. , Fidalgo, C. , Stancampiano, R. , Björklund, A. , & Carta, M. (2014). The anti‐dyskinetic effect of dopamine receptor blockade is enhanced in parkinsonian rats following dopamine neuron transplantation. Neurobiology of Disease, 62, 233–240. 10.1016/j.nbd.2013.09.021 24135006

[bph15145-bib-0068] Sprouse, J. S. , & Aghajanian, G. K. (1988). Responses of hippocampal pyramidal cells to putative serotonin 5‐HT1A and 5‐HT1B agonists: A comparative study with dorsal raphe neurons. Neuropharmacology, 27(7), 707–715. 10.1016/0028-3908(88)90079-2 2901680

[bph15145-bib-0069] Tseng, K. Y. , Riquelme, L. A. , Belforte, J. E. , Pazo, J. H. , & Murer, M. G. (2000). Substantia nigra pars reticulata units in 6‐hydroxydopamine‐lesioned rats: Responses to striatal D2 dopamine receptor stimulation and subthalamic lesions. The European Journal of Neuroscience, 12(1), 247–256. 10.1046/j.1460-9568.2000.00910.x 10651879

[bph15145-bib-0070] VanderMaelen, C. P. , Matheson, G. K. , Wilderman, R. C. , & Patterson, L. A. (1986). Inhibition of serotonergic dorsal raphe neurons by systemic and iontophoretic administration of buspirone, a non‐benzodiazepine anxiolytic drug. European Journal of Pharmacology, 129(1–2), 123–130. 10.1016/0014-2999(86)90343-2 2876903

[bph15145-bib-0071] Vegas‐Suarez, S. , Paredes‐Rodriguez, E. , Aristieta, A. , Lafuente, J. V. , Miguelez, C. , & Ugedo, L. (2019). Dysfunction of serotonergic neurons in Parkinson's disease and dyskinesia. International Review of Neurobiology, 146, 259–279. 10.1016/bs.irn.2019.06.013 31349930

[bph15145-bib-0072] Wang, Q. , Chen, J. , Li, M. , Lv, S. , Xie, Z. , Li, N. , … Zhang, W. (2019). Eltoprazine prevents levodopa‐induced dyskinesias by reducing causal interactions for theta oscillations in the dorsolateral striatum and substantia nigra pars reticulate. Neuropharmacology, 148, 1–10. 10.1016/j.neuropharm.2018.12.027 30612008

[bph15145-bib-0073] Wang, S. , Zhang, Q. J. , Liu, J. , Wu, Z. H. , Ali, U. , Wang, Y. , … Gui, Z. H. (2009a). The firing activity of pyramidal neurons in medial prefrontal cortex and their response to 5‐hydroxytryptamine‐1A receptor stimulation in a rat model of Parkinson's disease. Neuroscience, 162(4), 1091–1100. 10.1016/j.neuroscience.2009.04.069 19410634

[bph15145-bib-0074] Wang, S. , Zhang, Q. J. , Liu, J. , Wu, Z. H. , Wang, T. , Gui, Z. H. , … Wang, Y. (2009b). Unilateral lesion of the nigrostriatal pathway induces an increase of neuronal firing of the midbrain raphe nuclei 5‐HT neurons and a decrease of their response to 5‐HT1A receptor stimulation in the rat. Neuroscience, 159(2), 850–861. 10.1016/j.neuroscience.2008.12.051 19174182

[bph15145-bib-0075] Weiss, D. , Walach, M. , Meisner, C. , Fritz, M. , Scholten, M. , Breit, S. , … Krüger, R. (2013). Nigral stimulation for resistant axial motor impairment in Parkinson's disease? A randomized controlled trial. Brain, 136(7), 2098–2108. 10.1093/brain/awt122 23757762 PMC3692032

