# Supplementary material for: 6‐Hydroxydopamine lesion and levodopa treatment modify the effect of buspirone in the substantia nigra pars reticulata
Source: Br J Pharmacol. 2020 Jul 6;177(17):3957–74. doi: 10.1111/bph.15145 (PMC7429490; doi:10.1111/bph.15145)
Supplement: Supplementary file 1 — Figure S1. Validation of 6‐OHDA lesion and L‐DOPA induced abnormal involuntary movements. Evolution of dyskinesia scores showing (A) the time course of AIM scores for axial, limb and orolingual ratings and (B) locomotive score, on the last session after L‐DOPA chronic treatment. Results are expressed as means ± S.E.M. Coronal sections (C) from sham group and 6‐OHDA group. Note the lack of TH immunoreactivity in the striatum and SN. Scale bar = 1 mm. [file BPH-177-3957-s001.pdf]

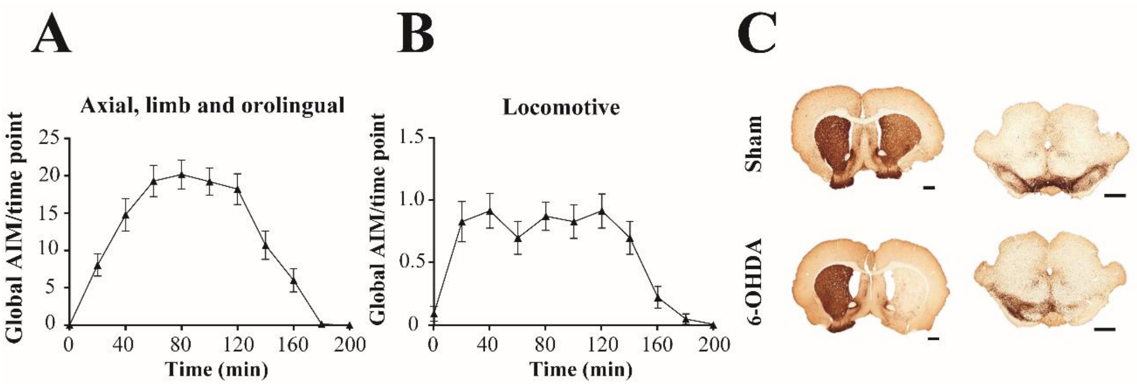

Supplementary Figure 1. Validation of 6-OHDA lesion and L-DOPA induced abnormal involuntary movements. Evolution of dyskinesia scores showing (A) the time course of AIM scores for axial, limb, and orolingual ratings and (B) locomotive score, on the last session after L-DOPA chronic treatment. Results are expressed as means ± S.E.M. Coronal sections (C) from sham group and 6-OHDA group. Note the lack of TH immunoreactivity in the striatum and SN. Scale bar = 1 mm.
