# Supplementary material for: 6‐Hydroxydopamine lesion and levodopa treatment modify the effect of buspirone in the substantia nigra pars reticulata
Source: Br J Pharmacol. 2020 Jul 6;177(17):3957–74. doi: 10.1111/bph.15145 (PMC7429490; doi:10.1111/bph.15145)
Supplement: Supplementary file 2 — Figure S2. Effect of buspirone on SNr spike trains and oscillatory activity of the SNr and the cortex. On the left, a recording track containing SNr spikes, SNr‐LFP and ECoG from the basal neuron‐firing pattern in the sham, 6‐OHDA and 6‐OHDA group. On the right, a recording track containing SNr spikes, SNr‐LFP and ECoG from the same neurons after buspirone administration (2.5 mg/kg, i.v.). Note that buspirone did not alter burst‐exhibiting pattern in sham group while buspirone reduce it in 6‐OHDA and 6‐OHDA L‐DOPA groups. [file BPH-177-3957-s002.pdf]

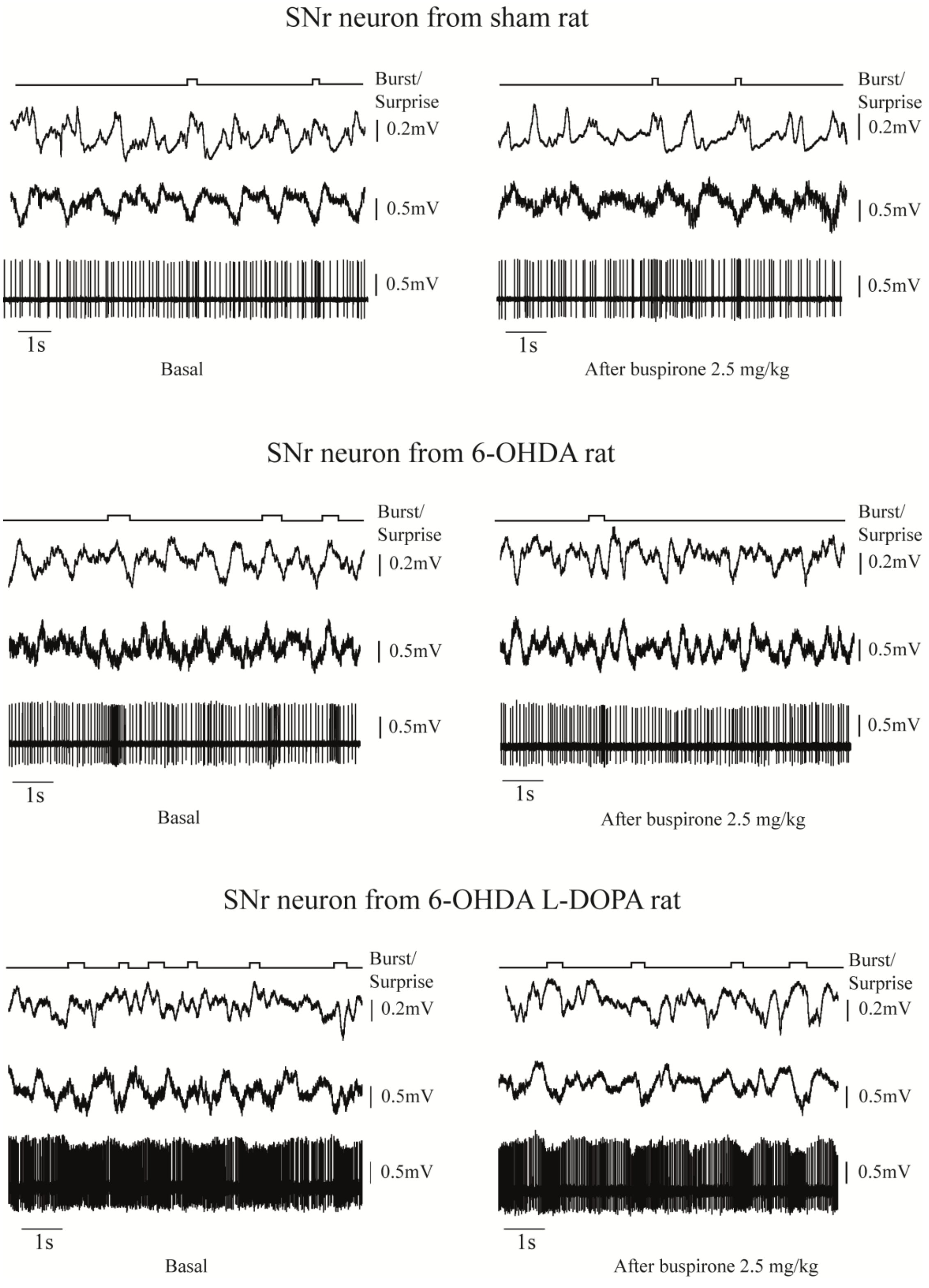

Supplementary Figure 2. Effect of buspirone on SNr spike trains and oscillatory activity of the SNr and the cortex. On the left, a recording track containing SNr spikes, SNr-LFP and ECoG from the basal neuron-firing pattern in the sham, 6-OHDA and 6-OHDA group. On the right, a recording track containing SNr spikes, SNr-LFP and ECoG from the same neurons after buspirone administration (2.5 mg/kg, i.v.). Note that buspirone did not alter burst-exhibiting pattern in sham group while buspirone reduce it in 6-OHDA and 6-OHDA L-DOPA groups.
