# Supplementary material for: 6‐Hydroxydopamine lesion and levodopa treatment modify the effect of buspirone in the substantia nigra pars reticulata
Source: Br J Pharmacol. 2020 Jul 6;177(17):3957–74. doi: 10.1111/bph.15145 (PMC7429490; doi:10.1111/bph.15145)
Supplement: Supplementary file 3 — Table S1. Statistical Details [file BPH-177-3957-s005.pdf]

Table S1. Statistical Details

One-way ANOVA		
	**F(DFn, DFd)**	**P value**
**Firing rate**	$F_{(2, 51)} = 3.738$	$p < 0.05$
**Coefficient of Variation**	$F_{(2, 51)} = 4.111$	$p < 0.05$
**ECoG Power spectrum (AUC values)**	$F_{(2, 51)} = 1.86$	n.s.
**LFP Power spectrum (AUC values)**	$F_{(2, 51)} = 1.86$	$p < 0.05$
**Coherence EcoG-SNr spikes (AUC values)**	$F_{(2, 51)} = 9.351$	$p < 0.05$
**Coherence LFP-SNr spikes (AUC values)**	$F_{(2, 51)} = 12.52$	$p < 0.05$
**Coherence ECoG-LFP (AUC values)**	$F_{(2, 51)} = 1.469$	n.s.
**Nigral GABA basal levels**	$F_{(2, 27)} = 0.123$	n.s.
**Nigral GLU basal levels**	$F_{(2, 27)} = 1.95$	n.s.
**SERT expression**	$F_{(2, 12)} = 10.14$	$p < 0.05$
**5-HT_{1A} expression**	$F_{(2, 12)} = 32.77$	$p < 0.05$

RM two-way ANOVA<br>F(DFn, DFd), p value		
	**Group**	**Drug**
**Local administration of buspirone**		
**Firing Rate**	$F_{(2, 13)} = 3.38$	$F_{(4, 52)} = 48.74$*
**Sistemic administration of buspirone**		
**Firing Rate**	$F_{(2, 20)} = 1.158$	$F_{(4, 80)} = 2.043$
**Coefficient of Variation**	$F_{(2, 20)} = 1.209$	$F_{(4, 80)} = 0.852$
**% effect on ECoG**	$F_{(2, 20)} = 0.339$	$F_{(4, 80)} = 9.816$*
**% effect on LFP**	$F_{(2, 20)} = 0.027$	$F_{(4, 80)} = 4.034$*
**% effect on coherence EcoG/SNr spikes**	$F_{(2, 20)} = 1.785$	$F_{(4, 80)} = 0.197$
**% effect on coherence LFP/SNr spikes**	$F_{(2, 20)} = 0.178$	$F_{(4, 80)} = 2.633$*

**% effect on coherence ECoG/LFP**	$F_{(2, 20)} = 0.038$	$F_{(4, 80)} = 5.718$*
**AUC (Net area) of GABA**	$F_{(2, 24)} = 6.408$*	$F_{(15, 360)} = 2.611$*
**AUC (Net area) of GLU**	$F_{(2, 23)} = 5.654$*	$F_{(15, 345)} = 3.009$*
**Sistemic administration of 8-OHDPAT**		
**Firing Rate**	$F_{(2, 12)} = 5.917$*	$F_{(4, 48)} = 5.467$*
**Coefficient of Variation**	$F_{(2, 12)} = 1.22$	$F_{(4, 48)} = 1.127$
**% effect on ECoG**	$F_{(2, 12)} = 1.231$	$F_{(4, 48)} = 1.04$
**% effect on LFP**	$F_{(2, 12)} = 0.228$	$F_{(4, 48)} = 2.37$
**% effect on coherence EcoG/SNr spikes**	$F_{(2, 12)} = 0.027$	$F_{(4, 48)} = 2.977$*
**% effect on coherence LFP/SNr spikes**	$F_{(2, 12)} = 0.454$	$F_{(4, 48)} = 1.1$
**% effect on coherence ECoG/LFP**	$F_{(2, 12)} = 2.609$*	$F_{(4, 48)} = 2.796$*
