# Supplementary material for: 6‐Hydroxydopamine lesion and levodopa treatment modify the effect of buspirone in the substantia nigra pars reticulata
Source: Br J Pharmacol. 2020 Jul 6;177(17):3957–74. doi: 10.1111/bph.15145 (PMC7429490; doi:10.1111/bph.15145)
Supplement: Supplementary file 4 — Table S2. Effect of buspirone on firing properties of SNr neurons. [file BPH-177-3957-s003.pdf]

Supplementary Table 2. Effect of buspirone on firing properties of SNr neurons.

Sham	Buspirone (mg/kg) i.v.					WAY-101635 (mg/kg) i.v.	
	Basal	0.6125	1.25	2.5	5	0.5	1
**Firing rate (Hz)**	23.0 ± 4.2	23.5 ± 4.5	23.3 ± 4.6	22.6 ± 5.0	23.4 ± 5.2	20.7 ± 5.9	18.1 ± 5.8
**Coefficient of variation**	27.6 ± 4.7	29.1 ± 4.1	31.7 ± 4.2	34.3 ± 5.1	29.1 ± 2.5	39.0 ± 7.9	45.1 ± 18.4
**Neurons exhibiting burst firing pattern (%)**	50	75[&]	50	50	62.5	50	40
**Number of bursts**	4.4 ± 2.6	1.7 ± 0.6	3.4 ± 1.5	7.3 ± 4.1	7.4 ± 4.6	5.0 ± 4.4	5.2 ± 3.4
**Duration of burst (ms)**	0.5 ± 0.3	0.4 ± 0.2	0.5 ± 0.2	0.8 ± 0.4	0.7 ± 0.4	5.0 ± 4.4	0.6 ± 0.6
**Spikes/burst**	9.9 ± 6.5	8.3 ± 3.7	9.5 ± 4.9	11.3 ± 5.0	9.0 ± 3.6	1.0 ± 0.9	18.3 ± 16.0
**Recurrence of burst (burst/min)**	3.0 ± 1.8	1.1 ± 0.4	2.2 ± 1.0	4.6 ± 2.6	4.6 ± 2.8	3.0 ± 2.2	3.2 ± 2.1
**Intraburst frequency (spike/s)**	27.5 ± 12.7	38.0 ± 13.5	20.6 ± 10.1	23.3 ± 12.8	41.4 ± 16.4	36.1 ± 17.9	20.6 ± 14.5

6-OHDA	Buspirone (mg/kg) i.v.					WAY-101635 (mg/kg) i.v.	
	Basal	0.6125	1.25	2.5	5	0.5	1
**Firing rate (Hz)**	30.1 ± 4.2	26.3 ± 6.0	31.6 ± 4.3	28.1 ± 3.9	28.1 ± 3.9	28.9 ± 5.3	26.6 ± 4.1
**Coefficient of variation**	44.3 ± 9.5	37.8 ± 6.2	38.5 ± 6.2	38.7 ± 6.9	42.8 ± 10.0	48.9 ± 18.3	48.6 ± 13.6
**Neurons exhibiting burst firing pattern (%)**	85.7$^{\$}$	71.4	57.1$^{\&}$	57.1$^{\&}$	42.9$^{\&}$	57.1$^{\&}$	33.3$^{\&}$
**Number of bursts**	9.0 ± 4.1	7.0 ± 3.8	6.3 ± 3.3	4.4 ± 2.3	4.1 ± 2.5	7.9 ± 3.9	6.8 ± 5.0
**Duration of burst (ms)**	0.2 ± 0.1	0.1 ± 0.1	0.1 ± 0.0	0.3 ± 0.1	0.5 ± 0.3	0.6 ± 0.2	0.2 ± 0.1
**Spikes/burst**	7.7 ± 2.2	7.4 ± 2.5	5.8 ± 2.4	11.2 ± 5.1	25.6 ± 18.9	18.4 ± 7.8	5.9 ± 3.7
**Recurrence of burst (burst/min)**	5.2 ± 2.8	4.4 ± 2.3	4.0 ± 2-0	2.9 ± 1.5	2.7 ± 1.7	5.2 ± 2.6	4.6 ± 3.3
**Intraburst frequency (spike/s)**	60.3 ± 12.2	53.0 ± 17.2	44.7 ± 17.2	31.2 ± 12.1	25.6 ± 13.9	21.4 ± 8.8	11.0 ± 7.3

6-OHDA L-DOPA	Buspirone (mg/kg) i.v.					WAY-101635 (mg/kg) i.v.	
	**Basal**	**0.6125**	**1.25**	**2.5**	**5**	**0.5**	**1**
**Firing rate (Hz)**	27.4 ± 3.4	37.5 ± 6.4	33.9 ± 6.6	31.1 ± 6.7	30.7 ± 6.3	29.5 ± 6.1	36.3 ± 8.1
**Coefficient of variation**	30.41 ± 5.2	37.0 ± 5.9	44.3 ± 8.3	47.4 ± 9.3	39.1 ± 7.2	38.6 ± 7.7	31.9 ± 6.9
**Neurons exhibiting burst firing pattern (%)**	$75^{\$}$	$37.5^{\&}$	$50^{\&}$	$50^{\&}$	$50^{\&}$	$50^{\&}$	$12.5^{\&}$
**Number of bursts**	19.2 ± 16.2	21.5 ± 16.6	28.5 ± 16.6	30.5 ± 16.2	31.2 ± 19.5	28.2 ± 20.8	16.3
**Duration of burst (ms)**	0.1 ± 0.1	0.1 ± 0.0	0.2 ± 0.1	0.2 ± 0.1	0.1 ± 0.0	0.2 ± 0.1	0.025
**Nº spikes/burst**	9.5 ± 3.7	5.9 ± 3.6	11.7 ± 4.4	8.5 ± 3.5	7.0 ± 3.2	10.4 ± 5.0	3.4
**Recurrence of burst (nº burst/min)**	11.7 ± 9.7	13.2 ± 9.9	17.6 ± 10.0	18.8 ± 9.7	18.9 ± 11.7	17.3 ± 12.4	9.8
**Intraburst frequency (spike/s)**	82.5 ± 26.8	49.7 ± 28.4	52.7 ± 26.5	53.6 ± 27.5	52.9 ± 27.2	57.5 ± 25.8	23.2

Data from the sham group (n = 8), 6-OHDA group (n = 7), and 6-OHDA L-DOPA group (n = 8) expressing the firing rate, coefficient of variation, percentage of neurons exhibiting burst firing pattern and burst related parameters defined as number of bursts, mean duration of the burst, mean number of spikes per burst, the recurrence of burst per min, and intraburst frequency or the interval of spike patterns within bursts. Burst activity was detected in the spike train in a period of 90s based following Surprise method. $^{\&}p < 0.05$ vs baseline and $^{\$}p < 0.05$ vs sham (Fisher´s exact test).
